# The chromatin remodeling subunit Baf200 promotes normal hematopoiesis and inhibits leukemogenesis

**DOI:** 10.1186/s13045-018-0567-7

**Published:** 2018-02-26

**Authors:** Lulu Liu, Xiaoling Wan, Peipei Zhou, Xiaoyuan Zhou, Wei Zhang, Xinhui Hui, Xiujie Yuan, Xiaodan Ding, Ruihong Zhu, Guangxun Meng, Hui Xiao, Feng Ma, He Huang, Xianmin Song, Bin Zhou, Sidong Xiong, Yan Zhang

**Affiliations:** 1 Institute of Biology and Medical Sciences, Soochow University, No. 199 Ren’ai Rd, Suzhou, China; 2 Key Laboratory of Molecular Virology and Immunology, Institut Pasteur of Shanghai, Chinese Academy of Sciences, 320 Yue-Yang Road, Shanghai, China; 3 University of Chinese Academy of Sciences, Beijing, China; 4 The State Key Laboratory of Cell Biology, CAS Center for Excellence in Molecular Cell Science, Shanghai Institute of Biochemistry and Cell Biology, Chinese Academy of Sciences, 320 Yue-Yang Road, Shanghai, China; 5 CAS-MPG Partner Institute for Computational Biology, Shanghai Institutes for Biological Sciences, Chinese Academy of Sciences, Shanghai, China; 6 Institute of Blood Transfusion, Chinese Academy of Medical Sciences and Peking Union Medical College, Chengdu, China; 7 School of Life Sciences, Shanghai University, Shanghai, China; 8 Bone Marrow Transplantation Center, the First Affiliated Hospital, Zhejiang University School of Medicine, Hangzhou, China; 9 Department of Hematology, Shanghai Jiao Tong University Affiliated Shanghai General Hospital, Shanghai, China

**Keywords:** Hematopoiesis, Leukemogenesis, Chromatin remodeling complex, Epigenetics

## Abstract

**Background:**

Adenosine triphosphate (ATP)-dependent chromatin remodeling SWI/SNF-like BAF and PBAF complexes have been implicated in the regulation of stem cell function and cancers. Several subunits of BAF or PBAF, including BRG1, BAF53a, BAF45a, BAF180, and BAF250a, are known to be involved in hematopoiesis. Baf200, a subunit of PBAF complex, plays a pivotal role in heart morphogenesis and coronary artery angiogenesis. However, little is known on the importance of Baf200 in normal and malignant hematopoiesis.

**Methods:**

Utilizing *Tie2-Cre*-, *Vav-iCre*-, and *Mx1-Cre*-mediated *Baf200* gene deletion combined with fetal liver/bone marrow transplantation, we investigated the function of Baf200 in fetal and adult hematopoiesis. In addition, a mouse model of MLL-AF9-driven leukemogenesis was used to study the role of Baf200 in malignant hematopoiesis. We also explored the potential mechanism by using RNA-seq, RT-qPCR, cell cycle, and apoptosis assays.

**Results:**

*Tie2-Cre*-mediated loss of *Baf200* causes perinatal death due to defective erythropoiesis and impaired hematopoietic stem cell expansion in the fetal liver. *Vav-iCre*-mediated loss of *Baf200* causes only mild anemia and enhanced extramedullary hematopoiesis. Fetal liver hematopoietic stem cells from *Tie2-Cre*^*+*^, *Baf200*^*f/f*^ or *Vav-iCre*^*+*^, *Baf200*^*f/f*^ embryos and bone marrow hematopoietic stem cells from *Vav-iCre*^*+*^, *Baf200*^*f/f*^ mice exhibited impaired long-term reconstitution potential in vivo. A cell-autonomous requirement of *Baf200* for hematopoietic stem cell function was confirmed utilizing the interferon-inducible *Mx1-Cre* mouse strain. Transcriptomes analysis revealed that expression of several erythropoiesis- and hematopoiesis-associated genes were regulated by Baf200. In addition, loss of *Baf200* in a mouse model of MLL-AF9-driven leukemogenesis accelerates the tumor burden and shortens the host survival.

**Conclusion:**

Our current studies uncover critical roles of Baf200 in both normal and malignant hematopoiesis and provide a potential therapeutic target for suppressing the progression of leukemia without interfering with normal hematopoiesis.

**Electronic supplementary material:**

The online version of this article (10.1186/s13045-018-0567-7) contains supplementary material, which is available to authorized users.

## Background

Hematopoiesis is a continuous process with a rare population of hematopoietic stem cells (HSCs) giving rise to all blood cell types. It is finely orchestrated by both cell-intrinsic factors and microenvironmental clues [1]. In mammals, hematopoiesis occurs sequentially during the development in the yolk sac (YS), aorta-gonad-mesonephros (AGM) region, placenta, fetal liver (FL), and bone marrow (BM). After birth, hematopoiesis continues with balanced proliferation, differentiation, quiescence, and apoptosis of HSCs. Various transcription factors, signaling pathways, and epigenetic regulators are involved in the regulation of these processes, and abnormalities occurring in these processes cause hematopoietic disorders including anemia or malignant transformation [2–11]. Therefore, defining factors involved in the regulation of hematopoiesis is an important issue in HSC biology.

Adenosine triphosphate (ATP)-dependent chromatin remodeling complexes include five different classes, SWI/SNF, ISWI, NuRD/Mi2/CHD, INO80, and SWR1, and are evolutionarily conserved. The SWI/SNF complexes of mammals include the BRG1-associated factor (BAF) complex and polybromo BRG1-associated factor (PBAF) complex. These two complexes consist of several common subunits and specific subunits. The common subunits include BRG1, BAF170, BAF155, BAF60a/b/c, BAF57, BAF53a/b, BAF47, BAF45a/b/c/d, and actin. BRM and BAF250a/b subunits are found only in the BAF complex; BAF200, BAF180, and BRD7 subunits are limited to the PBAF complex [12]. BRG1, BAF53a, BAF45a, BAF180, and BAF250a are known to be involved in hematopoiesis [13–22], but the hematopoietic activity of other BAF or PBAF subunits has not been determined.

BAF200, also known as ARID2, encoded by the *BAF200* gene, is a unique subunit of the PBAF chromatin remodeling complex, and inactivating mutations have been reported in a variety of human cancers [23–25]. *Baf200*-null mutant mice displayed embryonic lethality with multiple cardiac defects [26]. A recent study revealed a correlation between BAF200 defect and myelodysplasia [27]. However, the precise role of Baf200 in hematopoiesis has not been examined so far. Here, we investigated the function of *Baf200* in hematopoiesis through conditional deletion approach using the *Tie2-Cre*, *Vav-iCre*, and *Mx1-Cre* mice. *Tie2-Cre-*mediated loss of Baf200 causes embryonic lethality with defective fetal erythropoiesis, a reduced HSC pool in the FL, and impaired long-term reconstitution capacity of FL HSCs. *Vav-iCre*-mediated loss of Baf200 causes mild anemia and increased extramedullary hematopoiesis in adult mice. The long-term reconstitution potentials of the FL or BM HSCs from *Vav-iCre*^*+*^, *Baf200*^*f/f*^ mice are severely impaired*.* The loss of Baf200 alters the transcription of a cohort of genes involved in the maintenance of HSC homeostasis. In addition, *Baf200* deficiency accelerates the progression of MLL-AF9-induced leukemia. Taken together, the results demonstrate the involvement of Baf200 in both normal and malignant hematopoiesis and provide additional knowledge of the cellular and genetic activity of the chromatin remodeling complex in HSC function.

## Methods

### Mice

The *Baf200*^*LacZ/+*^ mice line was described previously [26]. *Baf200*^*LacZ/+*^ mice were crossed with *Actin-Flpe* transgenic mice to generate *Baf200*^*f/+*^ mice. Then, *Baf200*^*f/+*^ mice were further crossed with heterozygous *Tie2-Cre*, *Vav-iCre*, or *Mx1-Cre* transgenic mice to generate *Tie2-Cre*^*+*^, *Baf200*^*f/f*^, *Vav-iCre*^*+*^, *Baf200*^*f/f*^, or *Mx1-Cre*^*+*^, *Baf200*^*f/f*^ mice. All mice were bred under specific pathogen-free conditions. The protocols were approved by the Institutional Animal Care and Use Committee (IACUC) in Institut Pasteur of Shanghai. Genotyping and gene deletion efficiency were performed by polymerase chain reaction (PCR) using primers specific for wild-type (WT) *Baf200* alleles, floxed exon4 or deleted exon4. Gene deletion efficiency was also determined by reverse transcription-quantitative PCR (RT-qPCR) using primers in exon3 and exon4 (see Additional file 1: Table S2 and Table S3 for the primers).

### Flow cytometry

FL, BM, spleen, and thymus cells were isolated and passed through a 40-μm nylon cell strainer (BD Biosciences) and stained for 20 min on ice in PBS supplemented with 2% FBS. Dead cells were discarded from analysis by 4,6-diamino-2-phenylindole (DAPI) (Molecular Probes). All the antibodies used in the experiments are summarized in Additional file 1: Table S4. Flow cytometric analysis was performed on LSRII or Fortessa (BD Biosciences), and flow sorting was performed on FACSAriaII (BD Biosciences). Data were analyzed by FlowJo software (Tree Star, Ashland, OR).

### FL cell counting

Embryos were collected from female mice at days 12.5 to 17.5 of pregnancy, and the FLs dissected from each embryo were removed into 1 mL PBS supplemented with 2% FBS. To obtain single cells, the FLs were pipetted by 1 mL pipette gently and passed through a 40-μm nylon cell strainer (BD Biosciences). Then, the cell number was counted by hemocytometer.

### Transplantation assay

For competitive FL transplantation assay, E14.5 WT control and *Tie2-Cre*^*+*^, *Baf200*^*f/f*^ or *Vav-iCre*^*+*^, *Baf200*^*f/f*^ FL donor cells (CD45.2^+^) were isolated from timed pregnancies and single-cell suspensions were made using 40-μm nylon cell strainers (BD Biosciences). 2 × 10^6^ donor cells (CD45.2^+^) were mixed with 1 × 10^6^ C57BL/6 BM competitor cells (CD45.1^+^) prior to injection into lethally irradiated (9.5 Gy, X-ray) CD45.1^+^CD45.2^+^ C57BL/6 recipient mice. For competitive BM transplantation assay, 1 × 10^6^ WT or *Vav-iCre*^*+*^, *Baf200*^*f/f*^ BM donor cells (CD45.2^+^) were mixed with 1 × 10^6^ C57BL/6 BM competitor cells (CD45.1^+^) prior to injection into lethally irradiated (9.5 Gy, X-ray) CD45.1^+^CD45.2^+^ C57BL/6 recipient mice. Sixteen weeks later, the hematopoietic tissues were harvested and stained with antibodies for flow cytometry analysis to determine the ratios of donor- versus competitor-derived cells. Under the *Mx1-Cre* condition, 2 × 10^6^ WT or *Mx1-Cre*^*+*^, *Baf200*^*f/f*^ donor cells (CD45.2^+^ BM or FL) were mixed with 1 × 10^6^ C57BL/6 BM competitor cells (CD45.1^+^) prior to injection into lethally irradiated (9.5 Gy, X-ray) CD45.1^+^CD45.2^+^ C57BL/6 recipient mice, the recipient mice were bled 6–8 weeks after transplantation to analyze the percentage of chimerism in their blood and then injected with poly(I:C) (GE Healthcare Life Science) to induce the deletion of *Baf200* gene, and next, the reconstitution ability of donor cells was evaluated in the peripheral blood of recipient 1, 2, and 3 months after pIpC injection. For the assessment of the niche effect in hematopoiesis, 2 × 10^6^ control CD45.1^+^ BM cells were transplanted into lethally irradiated (9.5 Gy, X-ray) WT or *Vav-iCre*^*+*^, *Baf200*^*f/f*^ mice. Three months later, 6 × 10^6^ BM cells from recipient in the first transplant were mixed with 1 × 10^6^ CD45.1^+^CD45.2^+^ BM cells prior to injection into lethally irradiated (9.5 Gy, X-ray) CD45.2^+^ C57BL/6 recipient mice. For homing assay, 2 × 10^7^ FL cells from E14.5 WT or *Tie2-Cre*^*+*^, *Baf200*^*f/f*^ embryos or 2 × 10^7^ BM cells from WT or *Vav-iCre*^*+*^, *Baf200*^*f/f*^ mice labeled with 2 μM CellTrace Violet (Invitrogen) were transplanted into lethally irradiated CD45.2^+^ C57BL/6 recipient mice. Percentage of donor cells in the recipients was detected 16 h post-transplantation.

### Cell cycle and apoptosis analysis

For FL cell cycle analysis, pregnant female mice were injected intraperitoneally with BrdU for 1 h, and for BM LSK cell cycle analysis, WT or *Vav-iCre*^*+*^, *Baf200*^*f/f*^ mice were injected intraperitoneally with BrdU for 2 h. Then, FL or BM cells were isolated and stained with antibodies, followed by fixation, permeabilization, and stained with anti-BrdU antibody and Hoechst 33342 (Molecular Probes) according to the manufacturer’s instruction (BD Biosciences). For Ki67 staining, FL or BM cells were stained with surface markers to define subsets, followed by fixation, permeabilization, and stained with anti-Ki67 antibody and Hoechst 33342.

To detect apoptotic cells, FL or BM cells were stained with surface markers to define subsets, followed by staining with DAPI and anti-annexin V antibody in accordance with the manufacturer’s instruction (BD Biosciences).

### Histology

The FL, spleen, liver, and bone (femur and tibia) tissues were fixed in 4% paraformaldehyde overnight at room temperature. To decalcify, the bone tissues were placed into 10% EDTA solution and the solution was changed every other day for about 2 weeks. Then, the tissues were embedded in paraffin, sectioned at 6 μm, and stained with hematoxylin-eosin.

### Retroviral production, transformation, and leukemia mouse model

Retroviral supernatants were produced using HEK293T cells as described previously [28]. For leukemia cell production, Lin^−^ BM cells were isolated, pre-stimulated overnight, and infected with retrovirus. Infected cells were injected into lethally (9.5 Gy, X-ray) irradiated recipients. Leukemia cells were harvested from the sick mice and transplanted into sub-lethally (5 Gy, X-ray) irradiated recipients in secondary transplantation.

### RNA-seq and RT-qPCR

Total RNA was isolated from flow-sorted LSK cells or S3 cells using the RNeasy Mini kit (QIAGEN, Valencia, CA), and the RNA-seq analysis was performed at BGI (The Beijing Genomics Institute) via Illumina HiSeq™ 2000. Briefly, the mRNA was enriched by using the oligo (dT) magnetic beads, then fragmented into fragments. Then, the double-stranded complementary DNA (cDNA) was synthesized and purified by magnetic beads. End reparation and 3′-end single nucleotide A (adenine) addition was then preformed. Finally, sequencing adaptors were ligated to the fragments. The fragments were enriched by PCR amplification, and the quality of the library was assessed by using Agilent 2100 Bioanalyzer and ABI StepOnePlus Real-Time PCR system. Then, the library was ready for sequencing. Raw data were deposited in the Sequence Read Archive database (accession number SRP117796). Functional Gene Ontology analysis of differential genes was determined by using DAVID Bioinformatics Resources 6.8 [29, 30].

For RT-qPCR analysis of gene expression in LSK cells, cDNA was produced by EZ-press Cell to cDNA Kit (EZbioscience). For RT-qPCR analysis of gene expression in other cells, total RNA was isolated by TRIzol (invitrogen) and converted into cDNA by using FastKing RT Kit (Tiangen). Primers for PCR were designed using Primer3 software or selected from PrimerBank database (Additional file 1: Table S3). All the reactions were performed at ABI Prism 7900HT (Applied Biosystems) using SYBR® Premix Ex Taq ™ II (Takara).

### Statistical analysis

The significance of differences was determined with two-tailed paired Student’s *t* tests by using Prism software (GraphPad Software). Survival data from leukemia mouse models were analyzed by using a log-rank nonparametric test, and the data were expressed as Kaplan-Meier survival curves. Statistical significance is indicated by *p < 0.05, **p < 0.01, or ***p < 0.001.

## Results

### *Tie2-Cre*^*+*^, *Baf200*^*f/f*^ mice display embryonic lethality with defective fetal erythropoiesis

**Fig. 1:**
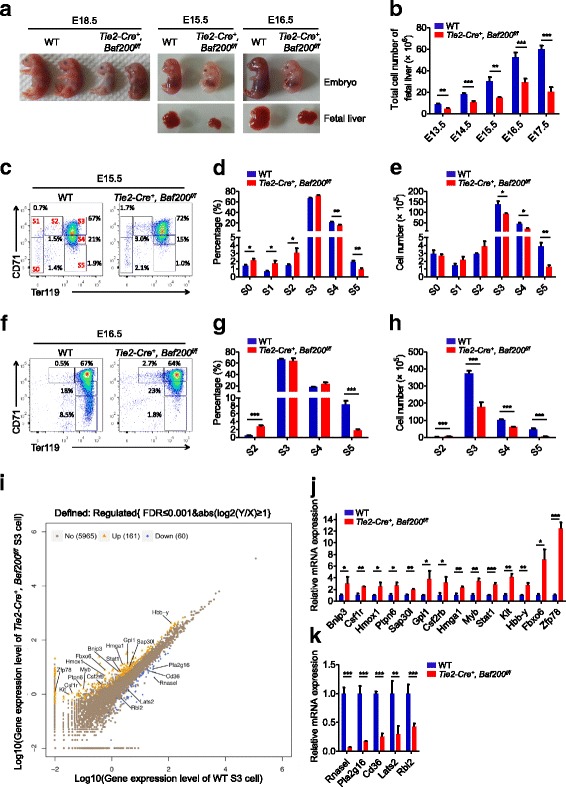
*Tie2-Cre*^*+*^, *Baf200*^*f/f*^ mice display embryonic lethality with defective fetal erythropoiesis. **a** Representative photographs of embryos and FLs from *Tie2-Cre*^*+*^, *Baf200*^*f/f*^ mice and WT littermate at E15.5, E16.5, and E18.5. **b** Absolute cell number of FLs from *Tie2-Cre*^*+*^, *Baf200*^*f/f*^ embryos and WT littermate at different developmental stages (*n* = 5–13 per genotype for each stages). **c** FACS profiles of CD71/Ter119 erythroid subsets in the FLs from E15.5 *Tie2-Cre*^*+*^, *Baf200*^*f/f*^ embryos and WT littermates. **d**, **e** Percentage (**d**) and absolute cell number (**e**) of CD71/Ter119 erythroid subsets from E15.5 *Tie2-Cre*^*+*^, *Baf200*^*f/f*^ embryos and WT littermates (*n* = 4 per genotype). **f** FACS profiles of CD71/Ter119 erythroid subsets in the FLs from E16.5 *Tie2-Cre*^*+*^, *Baf200*^*f/f*^ embryos and WT littermates. **g**, **h** Percentage (**g**) and absolute cell number (**h**) of CD71/Ter119 erythroid subsets from E16.5 *Tie2-Cre*^*+*^, *Baf200*^*f/f*^ embryos and WT littermates (*n* = 4 per genotype). **i** Scatter plots of all expressed genes in S3 cells from WT and *Tie2-Cre*^*+*^, *Baf200*^*f/f*^ FLs. Blue indicates downregulation gene, yellow indicates upregulation gene, and gray indicates non-regulation gene in *Tie2-Cre*^*+*^, *Baf200*^*f/f*^ S3 cells. **j**, **k** Upregulation genes (**j**) and downregulation genes (**k**) in *Tie2-Cre*^*+*^, *Baf200*^*f/f*^ S3 cells were confirmed by RT-qPCR (*n* = 3 per genotype). Data are shown as means ± SEM. *P < 0.05; **P < 0.01; ***P < 0.001

The expression of *Baf200* was examined in representative hematopoietic lineages. *Baf200* is widely expressed in all hematopoietic lineages and particularly in FL LSK (Lin^−^Sca1^+^c-Kit^+^), FL hematopoietic progenitor cell (HPC) (Lin^−^Sca1^−^c-Kit^+^), FL S2 (Ter119^low^CD71^+^), FL S3 (Ter119^high^CD71^+^), adult BM short-term (ST)-HSC (Lin^−^Sca1^+^c-Kit^+^CD34^+^Flt3^low^), multipotent progenitor (MPP) (Lin^−^Sca1^+^c-Kit^+^CD34^+^Flt3^+^), megakaryocyte–erythroid progenitor (MEP) (Lin^−^Sca1^−^c-Kit^+^CD34^−^CD16/32^low^), and granulocyte-macrophage progenitor (GMP) (Lin^−^Sca1^−^c-Kit^+^CD34^+^CD16/32^high^) lineages (Additional file 1: Figure S1). To investigate its role in hematopoiesis in vivo, *Baf200* was conditionally deleted by crossing *Baf200*^*f/f*^ mice with *Tie2-Cre* transgenic mice which express Cre-recombinase in endothelial cells and hematopoietic lineages [31]. Only four *Tie2-Cre*^*+*^, *Baf200*^*f/f*^ mice were obtained among 239 offspring from intercross between male *Tie2-Cre*^*+*^, *Baf200*^*f/+*^ mice and female *Baf200*^*f/f*^ mice at weaning age, which was considerably lower than the expected Mendelian ratio (Additional file 1: Figure S2a). The four *Tie2-Cre*^*+*^, *Baf200*^*f/f*^ mice exhibited low recombination efficiency of the LoxP sites in hematopoietic cells (data not shown). Examination of the genotypes in the litters from timed mating intercross found that *Tie2-Cre*^*+*^, *Baf200*^*f/f*^ embryos were alive and were recovered at the expected Mendelian ratio at embryonic day (E) 17.5, but that the majority died by E18.5 (Fig. 1a and Additional file 1: Figure S2a).

Genomic PCR or RT-qPCR confirmed the deletion of *Baf200* in the FL cells of E14.5 *Tie2-Cre*^*+*^, *Baf200*^*f/f*^ embryos (Additional file 1: Figure S2c, d), suggesting that *Baf200* was efficiently deleted in the hematopoietic lineages. *Tie2-Cre*^*+*^, *Baf200*^*f/f*^ embryos showed severe anemia and profound growth retardation as early as E15.5 compared with their WT *Tie2-Cre*^*−*^, *Baf200*^*f/f*^ or *Tie2-Cre*^*−*^, *Baf200*^*f/+*^ littermates (Fig. 1a and Additional file 1: Figure S2b). The absolute cell number of FL from *Tie2-Cre*^*+*^, *Baf200*^*f/f*^ embryos was also reduced since E13.5 (Fig. 1b). The apoptosis of FL cells in *Tie2-Cre*^*+*^, *Baf200*^*f/f*^ and WT embryos at E14.5 and E16.5 was measured using annexin-V and DAPI staining followed by flow cytometric analysis. Although the apoptosis of FL cells were comparable between *Tie2-Cre*^*+*^, *Baf200*^*f/f*^ and WT embryos at E14.5, the percentage of annexin-V^+^, DAPI^−^ and annexin-V^+^, DAPI^+^ FL cells in *Tie2-Cre*^*+*^, *Baf200*^*f/f*^ embryos at E16.5 is significantly higher than that in WT embryos (Additional file 1: Figure S2e). These results suggested that increased apoptosis might account, at least in part, for the reduced FL cell number in *Tie2-Cre*^*+*^, *Baf200*^*f/f*^ embryos.

Evaluation of erythropoiesis in the FL from WT and *Tie2-Cre*^*+*^, *Baf200*^*f/f*^ embryos by staining with Ter119 and CD71 surface markers revealed that the percentages of S0, S1, and S2 cells were increased and the percentages of S4 and S5 cells were decreased in E15.5 *Tie2-Cre*^*+*^, *Baf200*^*f/f*^ FL (Fig. 1c, d). Although the total number of FL cells in the *Tie2-Cre*^*+*^, *Baf200*^*f/f*^ embryos was reduced, the absolute numbers of S0, S1, and S2 cells remained unchanged, whereas the absolute numbers of S3, S4, and S5 cells were reduced (Fig. 1e). Notably, similar phenotype was also observed at E13.5 and E14.5 (Additional file 1: Figure S2f–g) and became much more apparent at E16.5 (Fig. 1f–h). Though the percentage of FL MEP (Lin^−^Sca-1^−^c-Kit^+^CD16/32^−^CD71^−^) was increased, the absolute cell number remained unchanged in E14.5 *Tie2-Cre*^*+*^, *Baf200*^*f/f*^ FL (Additional file 1: Figure S2 h**)**. We found that the cell cycle status and apoptosis of FL erythrocytes from *Tie2-Cre*^*+*^, *Baf200*^*f/f*^ embryos were comparable to those from WT embryos (Additional file 1: Figure S2i–k), suggesting that the defective erythropoiesis in *Tie2-Cre*^*+*^, *Baf200*^*f/f*^ FL was not caused by reduced cell proliferation or increased apoptosis. These results indicate that *Baf200* plays a pivotal role in the erythroid terminal differentiation in the FL.

To determine the potential mechanism by which Baf200 regulates fetal erythropoiesis, we performed RNA-seq analysis of the transcriptomes of S3 cells from E14.5 WT or *Tie2-Cre*^*+*^, *Baf200*^*f/f*^ embryos. A total 161 upregulated genes and 60 downregulated genes were observed in *Tie2-Cre*^*+*^, *Baf200*^*f/f*^ S3 cells (Fig. 1i). Functional Gene Ontology (GO) analysis of differentially expressed genes indicated significant enrichment in translation, proteolysis involved in cellular protein catabolic process, glycosphingolipid metabolic process, erythrocyte homeostasis, intracellular signal transduction, response to oxidative stress, and oxidation-reduction process terms (Additional file 1: Figure S3). During the transition from S2 to S3, the expression level of *Myb* and *Kit* is downregulated [32], and the downregulation of *Myb* is important for erythroid terminal differentiation [33, 34]. In addition, *Hmox1* overexpression in erythroid cells decreases hemoglobin synthesis [35]. Moreover, the expression of *Pla2g16* is induced during erythroid maturation, and *Pla2g16* deficiency leads to impaired proerythroblasts (ProE) terminal differentiation [36]. We found that the expression of *Myb*, *Hmox1*, and *Kit* was increased, while the expression *Pla2g16* was decreased in *Tie2-Cre*^*+*^, *Baf200*^*f/f*^ S3 cells (Fig. 1i–k).These may partially explain the impaired FL erythropoiesis in *Tie2-Cre*^*+*^, *Baf200*^*f/f*^ embryos.

### The expansion of FL HSCs in *Tie2-Cre*^*+*^, *Baf200*^*f/f*^ embryos is severely impaired

**Fig. 2:**
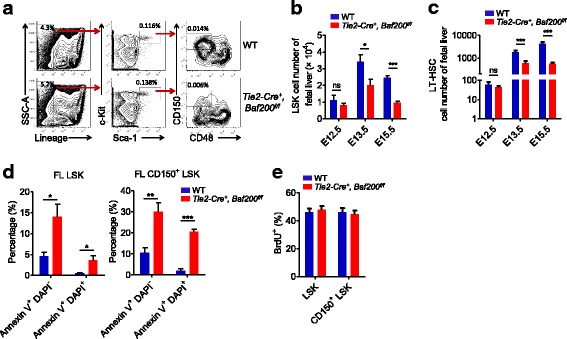
The expansion of FL HSCs in *Tie2-Cre*^*+*^, *Baf200*^*f/f*^ embryos is severely impaired. **a** FACS profiles of LSK and LT-HSC (CD150^+^CD48^−^LSK) subsets in the FLs from E15.5 *Tie2-Cre*^*+*^, *Baf200*^*f/f*^ embryos and WT littermates. **b**, **c** Absolute cell number of LSK cells (**b**) and LT-HSCs (**c**) from *Tie2-Cre*^*+*^, *Baf200*^*f/f*^ embryos and WT littermates at indicated stages. **d** Percentage of apoptotic cells of LSK and CD150^+^ LSK cells from E14.5 *Tie2-Cre*^*+*^, *Baf200*^*f/f*^ embryos (*n* = 4) and WT littermates (*n* = 5). The apoptosis was evaluated by using annexin V and DAPI. **e** Cell cycle status of LSK and CD150^+^ LSK cells from E14.5 *Tie2-Cre*^+^, *Baf200*^*f/f*^ embryos (*n* = 4) and WT littermates (*n* = 5). No difference of BrdU^+^ cells was observed. Data are shown as means ± SEM. *P < 0.05; **P < 0.01; ***P < 0.001. ns indicates no significant difference

We then examined the hematopoietic stem and progenitor cells in WT or *Tie2-Cre*^+^, *Baf200*^*f/f*^ FL (Fig. 2a). The absolute number of LSK (Lin^−^Sca1^+^c-Kit^+^) cells and long-term (LT)-HSCs (CD150^+^CD48^−^LSK) from *Tie2-Cre*^*+*^, *Baf200*^*f/f*^ FL was similar to those from WT FL at E12.5 (Fig. 2b, c). However, the absolute number of LSK cells and LT-HSC was significantly decreased in E13.5 and E15.5 *Tie2-Cre*^*+*^, *Baf200*^*f/f*^ FL (Fig. 2b, c), indicating that the expansion, but not the initial seeding, of HSCs in the FL was impaired in *Tie2-Cre*^*+*^, *Baf200*^*f/f*^ embryos. We next detected the cell proliferation and apoptosis of LSK and CD150^+^LSK cells from E14.5 *Tie2-Cre*^*+*^, *Baf200*^*f/f*^ embryos. There was a significant increase in apoptosis of the LSK and CD150^+^ LSK cells of *Tie2-Cre*^*+*^, *Baf200*^*f/f*^ embryos, while the cell proliferation remains unchanged (Fig. 2d, e). Hence, *Tie2-Cre*-mediated loss of Baf200 causes aberrant apoptosis of FL LSK and LT-HSC.

To further identify the downstream mediators of Baf200 in FL HSCs, we analyzed the transcriptomes of FL LSK cells from E14.5 WT or *Tie2-Cre*^*+*^, *Baf200*^*f/f*^ embryos by RNA-seq. A total of 94 genes were upregulated and 77 genes were downregulated in *Tie2-Cre*, *Baf200*^*f/f*^ FL LSK cells compared with WT cells (Additional file 1: Figure S4a). GO analysis of these 171 differentially expressed genes indicated significant enrichment in cell adhesion, reactive oxygen species metabolic process, inflammatory response, and phagocytosis pathway, as well as hemopoiesis (Additional file 1: Figure S4c). Alterations in the expression of hematopoiesis-associated genes, including *Hck*, *Csf1r*, *Procr*, *Gprasp2*, *Fstl1*, and *Tbxas1* [37–41], were confirmed by RT-qPCR (Additional file 1: Figure S4b).

### Baf200 is required for the FL HSC maintenance

**Fig. 3:**
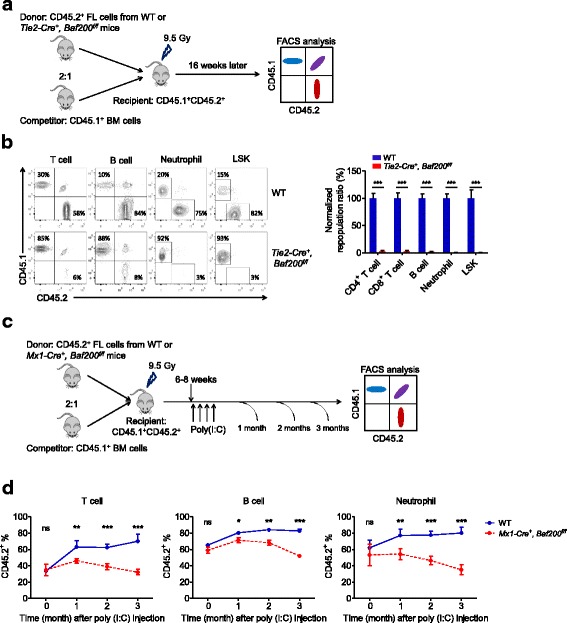
Baf200 is required for the FL HSC maintenance. **a** Scheme of competitive FL transplantation assay. **b** Representative FACS profiles showing the frequency of T cells (CD3^+^), B cells (B220^+^), neutrophils (Gr1^high^CD11b^+^) in the peripheral blood, and LSK (Lin^−^Sca1^+^c-Kit^+^) cells in the BM from recipient mice 16 weeks after transplantation (left) and the graph showing the relative ratios of CD45.2 versus CD45.1 of the indicated cell types in recipient mice 16 weeks after transplantation (right) (*n* = 5 per genotype). **c** Scheme of competitive FL transplantation assay in *Mx1-Cre* mouse strain. **d** Percentage of donor-derived T cells (left), B cells (middle), and neutrophils (right) in the peripheral blood from recipient mice assessed at different time points (*n* = 5 per genotype for each subset). Data are shown as means ± SEM. *P < 0.05; **P < 0.01; ***P < 0.001

A competitive repopulation assay was performed by co-transplanting FL cells from CD45.2^+^ *Tie2-Cre*^*+*^, *Baf200*^*f/f*^ embryos or CD45.2^+^ WT controls with CD45.1^+^ BM cells at a ratio of 2:1 into lethally irradiated CD45.1^+^CD45.2^+^ recipient mice (Fig. 3a). Reconstitution by donor cells was evaluated in the peripheral blood, spleen, thymus, and BM of the recipient mice 16 weeks post-transplantation. As expected, WT donor cells competed effectively with the competitor cells. In contrast, *Tie2-Cre*^*+*^, *Baf200*^*f/f*^ FL cells failed to contribute the long-term reconstitution of the myeloid, T, and B cell lineages or the LSK cells in the chimeras (Fig. 3b and Additional file 1: Figure S5a, b). The homing capacity of *Tie2-Cre*^*+*^, *Baf200*^*f/f*^ FL cells remains unchanged (Additional file 1: Figure S5c). We next generated an inducible *Baf200*-deletion mouse model by crossing *Mx1-Cre* with *Baf200*-flox mice. FL cells from *Mx1-Cre*^*+*^, *Baf200*^*f/f*^ (before deletion) or control embryos were transplanted together with competitor BM cells into lethally irradiated recipients. Six to 8 weeks later, the recipient mice received poly(I:C), and the peripheral blood was analyzed for 3 months (Fig. 3c). Deletion of *Baf200* led to gradual but significant decrease of lymphoid and myeloid lineages in the recipients (Fig. 3d). These results suggest that a cell-intrinsic role of Baf200 is required for FL HSC function.

### Baf200 contributes to adult erythropoiesis

**Fig. 4:**
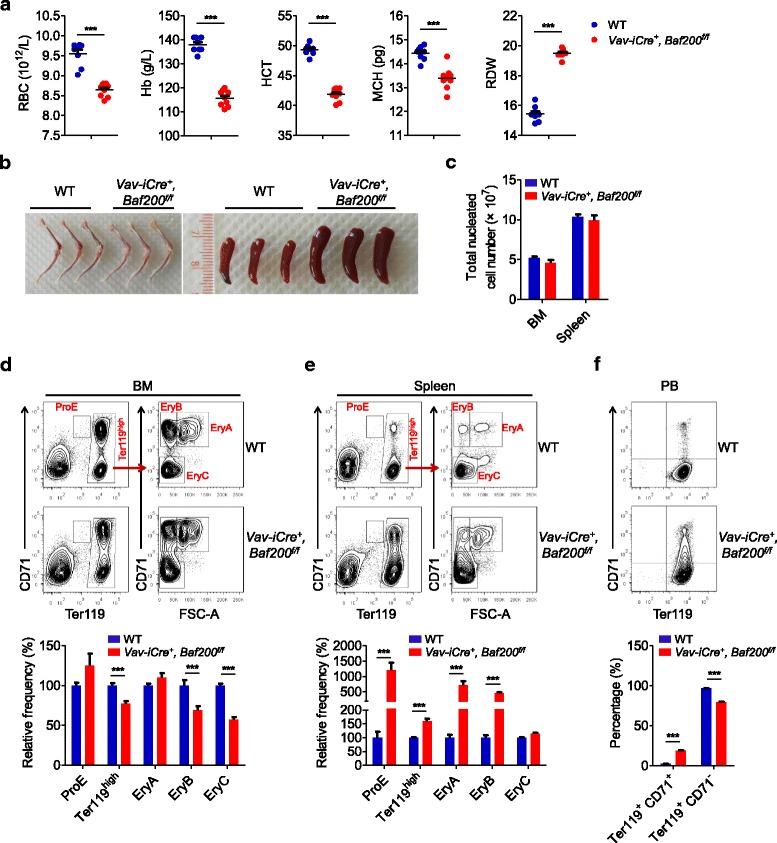
Baf200 contributes to adult erythropoiesis. **a** Red blood cell parameters of peripheral blood from 10- to 12-week-old WT (*n* = 8) and *Vav-iCre*^*+*^, *Baf200*^*f/f*^ (*n* = 9) mice. **b** Representative photographs of bone and spleen from 8-week-old WT and *Vav-iCre*^+^, *Baf200*^*f/f*^ mice. **c** Graph showing the total nucleated cell number of BM and spleen from 8-week-old WT and *Vav-iCre*^+^, *Baf200*^*f/f*^ mice (*n* = 6 per genotype). **d** Representative FACS profiles (top) and graph (bottom) showing relative frequency of CD71/Ter119 erythroid subsets in the BM from WT and *Vav-iCre*^*+*^, *Baf200*^*f/f*^ mice (*n* = 6 per genotype for each subset). **e** Representative FACS profiles (top) and graph (bottom) showing relative frequency of CD71/Ter119 erythroid subsets in the spleen from WT and *Vav-iCre*^*+*^, *Baf200*^*f/f*^ mice (*n* = 6 per genotype for each subset). **f** Representative FACS profiles (top) and graph (bottom) showing percentage of CD71/Ter119 erythroid subsets in the peripheral blood from WT and *Vav-iCre*^+^, *Baf200*^*f/f*^ mice (*n* = 10 per genotype for each subset). Data are shown as means ± SEM. ***P < 0.001

To further explore the in vivo function of Baf200 in hematopoiesis, we generated *Vav-iCre*^*+*^, *Baf200*^*f/f*^ mice by utilizing *Vav-iCre* mice strain which express *Cre*-recombinase in hematopoietic lineages [42]. The *Vav-iCre*^*+*^, *Baf200*^*f/f*^ mice were recovered at the expected Mendelian ratio and displayed an appearance indistinguishable from the *Vav-iCre*^*−*^, *Baf200*^*f/f*^ or *Vav-iCre*^*−*^, *Baf200*^*f/+*^ WT controls. Efficient deletion of *Baf200* in the BM, spleen, and thymus cells from *Vav-iCre*^*+*^, *Baf200*^*f/f*^ mice was confirmed by genomic PCR (Additional file 1: Figure S6a). Peripheral blood counts revealed that *Vav-iCre*^*+*^, *Baf200*^*f/f*^ mice exhibited decreased RBC number, hemoglobin concentration, hematocrit, and mean corpuscular hemoglobin along with increased RDW, suggesting a mild anemia may occur in *Vav-iCre*^*+*^, *Baf200*^*f/f*^ mice (Fig. 4a and Additional file 1: Figure S6b). Consistently, *Vav-iCre*^*+*^, *Baf200*^*f/f*^ mice exhibited slightly paler bone and splenomegaly (Fig. 4b). However, the total number of nucleated cells in the BM and spleen from *Vav-iCre*^*+*^, *Baf200*^*f/f*^ mice were comparable with those from WT mice (Fig. 4c). Flow cytometric analysis revealed impairment of erythropoiesis with decreased frequencies of EryB and EryC cells in the BM and increased frequencies of ProE and Ter119^high^ cells in the spleen from *Vav-iCre*^*+*^, *Baf200*^*f/f*^ mice (Fig. 4d, e). The activation of erythropoiesis in the spleen might have been an attempt to compensate for insufficient erythropoiesis in the BM. Surprisingly, an increase of immature erythrocytes defined as Ter119^+^ CD71^+^ and a decrease of mature erythrocytes defined as Ter119^+^CD71^−^ were detected in the peripheral blood of *Vav-iCre*^*+*^, *Baf200*^*f/f*^ mice (Fig. 4f). Taken together, the results indicate that *Baf200* contributes to adult erythropoiesis.

### Baf200 is involved in maintaining the adult hematopoiesis in steady state

**Fig. 5:**
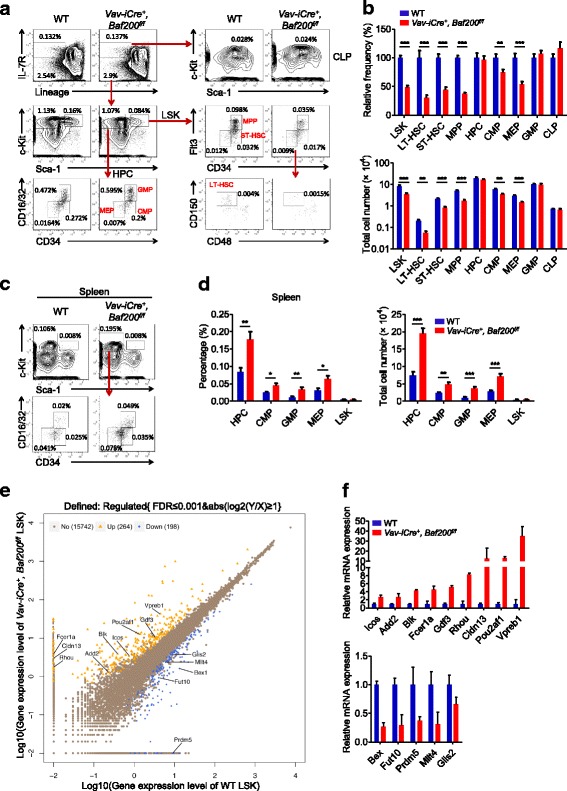
Baf200 is required for adult hematopoiesis in steady state. **a** Representative FACS profiles showing hematopoietic stem and progenitor cells (HSPCs) in the BM from WT and *Vav-iCre*^*+*^, *Baf200*^*f/f*^ mice. **b** Graph showing the relative frequency (top) and absolute cell number (bottom) of the indicated subsets in the BM from WT and *Vav-iCre*^*+*^, *Baf200*^*f/f*^ mice (*n* = 5–9 per genotype for each subset). **c** Representative FACS profiles showing hematopoietic progenitors in the spleen from male WT and *Vav-iCre*^*+*^, *Baf200*^*f/f*^ mice (8–10-week-old). **d** Graph showing the percentage (left) and absolute cell number (right) of the indicated subsets in the spleen from male WT and *Vav-iCre*^+^, *Baf200*^*f/f*^ mice (8–10-week-old) (*n* = 6 per genotype for each subset). **e** Scatter plots of all expressed genes in BM LSK cells from WT and *Vav-iCre*^*+*^, *Baf200*^*f/f*^ mice. Blue indicates downregulation gene, yellow indicates upregulation gene, and grey indicates non-regulation gene in *Vav-iCre*^+^, *Baf200*^*f/f*^ LSK cells. **f** Upregulation genes and downregulation genes in *Vav-iCre*^+^, *Baf200*^*f/f*^ LSK cells were confirmed by RT-qPCR (*n* = 2 per genotype). Data are shown as means ± SEM. *P < 0.05; **P < 0.01; ***P < 0.001

Flow cytometric analysis of the HSC compartments demonstrated decreases in the percentages and absolute numbers of LT-HSCs, ST-HSCs, MPP cells, common myeloid progenitor (CMP) cells, and MEP cells in the BM of *Vav-iCre*^*+*^, *Baf200*^*f/f*^ mice (Fig. 5a, b). Enhanced extramedullary hematopoiesis in the spleen usually accompanies a decrease in BM hematopoiesis. Indeed, the percentages and numbers of CMP, GMP, and MEP cells in the spleen of *Vav-iCre*^*+*^, *Baf200*^*f/f*^ mice were increased (Fig. 5c, d), indicating that *Baf200* deficiency promotes extramedullary hematopoiesis. Flow cytometric analysis found no significant differences in the cell cycle status or apoptosis of LSK cells in *Vav-iCre*^*+*^, *Baf200*^*f/f*^ mice and WT controls (Additional file 1: Figure S7a–g). The data reveal that Baf200 is not required for cell cycle process or apoptosis of the adult LSK compartment in steady-state hematopoiesis.

To determine the potential molecular pathways by which Baf200 regulates BM HSCs function, we conducted RNA-seq assay on purified BM LSK cells from *Vav-iCre*^*+*^, *Baf200*^*f/f*^ and WT mice. A total of 264 genes were upregulated and 198 genes were downregulated in *Baf200*-deficient LSK cells (Fig. 5e). Although the top GO terms are related to immune response, cell adhesion, and metabolic process, the erythrocyte development and hematopoiesis terms were also observed (Additional file 1: Figure S8). RT-qPCR experiment confirmed the altered expression of several hematopoiesis-associated genes (Fig. 5f), suggesting that Baf200 has an impact on adult HSC function, at least in part through regulation of the hematopoiesis-related genes.

### Cell-intrinsic Baf200 is required for HSC maintenance

**Fig. 6:**
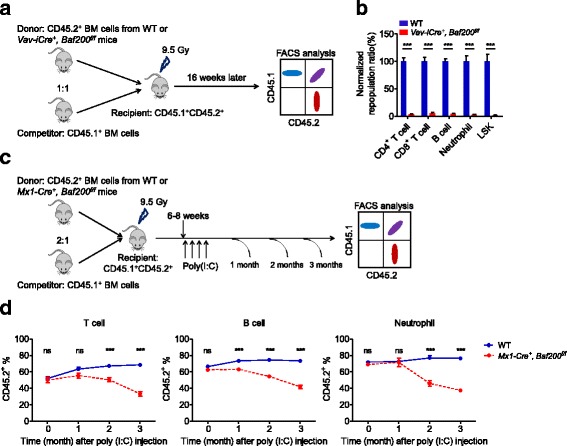
Baf200-deficient BM HSCs have a significantly impaired long-term reconstitution capacity. **a** Scheme of competitive BM transplantation assay for WT and *Vav-iCre*^*+*^, *Baf200*^*f/f*^ mice. **b** The graph showing the relative ratios of CD45.2 versus CD45.1 of the indicated cell types in recipient mice 16 weeks after transplantation (*n* = 7 per genotype for each subset). **c** Scheme of competitive BM transplantation assay for WT and *Mx1-Cre*^*+*^, *Baf200*^*f/f*^ mice. **d** Percentage of donor-derived T cells (left), B cells (middle), and neutrophils (right) in the peripheral blood from recipient mice assessed at different time point (*n* = 5 per genotype for each subset). Data are shown as means ± SEM. ***P < 0.001

Competitive hematopoietic reconstitution assays were performed to further characterize the requirement of *Baf200* for adult HSC maintenance by transplanting BM cells from *Vav-iCre*^*+*^, *Baf200*^*f/f*^ or WT controls together with competitor BM cells into lethally irradiated recipients (Fig. 6a). Sixteen weeks post-transplantation, *Baf200*-deficient HSCs failed to repopulate lymphoid and myeloid lineages in the peripheral blood, BM, spleen, and thymus of the recipients (Fig. 6b and Additional file 1: Figure S9a). Moreover, the mutant donor-derived LSK cells were barely detectable in chimeras despite the comparable homing capacity of WT and *Baf200*-deficient BM cells (Fig. 6b and Additional file 1: Figure S9b). The results indicate that *Baf200* is required for the maintenance of adult BM HSCs.

To further elucidate whether Baf200 regulates hematopoiesis in a cell-intrinsic manner, we firstly utilized inducible *Baf200*-deletion mice (*Mx1-Cre*^*+*^, *Baf200*^*f/f*^) and performed competitive transplantation experiments as above (Fig. 6c). Before poly(I:C) injection, *Mx1-Cre*^*+*^, *Baf200*^*f/f*^ and WT BM cells had similar reconstitution capacity, while loss of Baf200 also led to gradual but significant decrease of lymphoid and myeloid lineages in the recipients (Fig. 6d). In addition, we transplanted CD45.1^+^ BM cells into lethally irradiated *Vav-iCre*^*+*^, *Baf200*^*f/f*^ or WT mice. Two months later, BM cells from the recipients were harvested and co-transplanted into lethally irradiated recipients together with competitor cells. Sixteen weeks later, flow cytometry assays were performed to detect the lineage contribution of transplanted cells (Additional file 1: Figure S9c). The results revealed no difference in contribution ratios of any lineages (Additional file 1: Figure S9d). Therefore, these data demonstrate that *Baf200* is a cell autonomously required for the adult HSC maintenance.

Considering that *Tie2-Cre-*mediated deletion of *Baf200* led to embryonic lethality, we then detected the effects of *Baf200* deletion on fetal hematopoiesis from *Vav-iCre*^*+*^, *Baf200*^*f/f*^ mice. Although there was no difference in the absolute number of FL cells in E14.5 *Vav-iCre*^*+*^, *Baf200*^*f/f*^ and WT embryos (Additional file 1: Figure S10a), detailed flow cytomeric analysis showed that there was a blockade of S3 to S4 transition during erythroid differentiation in the *Vav-iCre*^+^, *Baf200*^*f/f*^ embryos (Additional file 1: Figure S10b). The frequency and total number of LSK cells and LT-HSCs in the FL from *Vav-iCre*^*+*^, *Baf200*^*f/f*^ embryos remained unchanged compared with those in the littermate controls (Additional file 1: Figure S10c). However, competitive transplantation assay demonstrated that FL cells from *Vav-iCre*^*+*^, *Baf200*^*f/f*^ embryos also showed impaired long-term reconstitution capacity (Additional file 1: Figure S10d–f).

### Loss of Baf200 accelerates leukemogenesis in MLL-AF9-induced AML

**Fig. 7:**
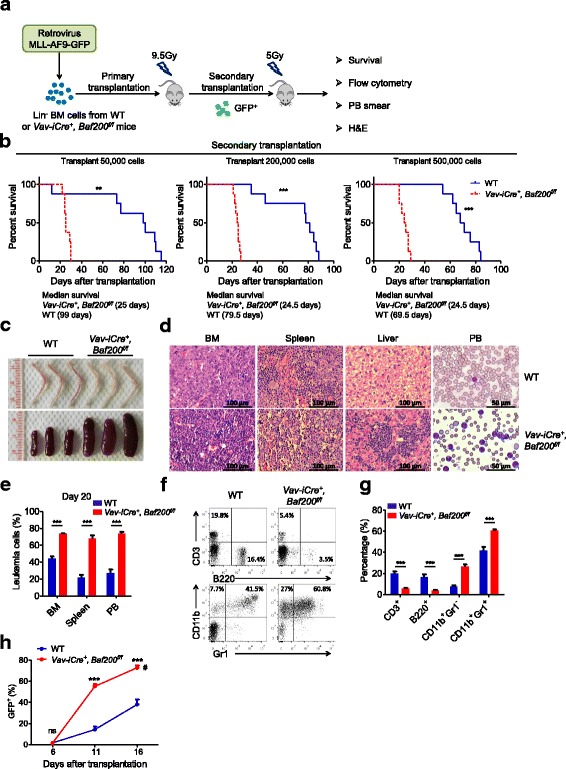
Loss of Baf200 accelerates leukemogenesis in MLL-AF9-induced AML. **a** Scheme to investigate the role of endogenous Baf200 in MLL-AF9-induced AML. **b** Survival curve of recipient mice engrafted with leukemia cells from primary recipients in the secondary transplantation assay (*n* = 8 per genotype). **c** Representative photos of bone and spleen from recipient mice. **d** Histology of the BM, spleen, and liver and blood smear from recipients. **e** Graph showing the percentage of leukemia cells in the BM (*n* = 3 per genotype), spleen (*n* = 3 per genotype), and PB (peripheral blood) (*n* = 8 per genotype) from recipient mice. **f** Representative flow profiles of myeloid and lymphoid subsets in the PB from recipients. **g** Graph showing the percentage of the indicated subsets in the PB (*n* = 8 per genotype for each subset). **h** Graph showing the percentage of leukemia cells (GFP^+^) in the PB from recipients at indicated days post-transplantation (*n* = 5 per genotype for each subset). *Vav-iCre*^+^, *Baf200*^*f/f*^ group mice showed accelerated accumulation of leukemia cells in the PB. The number sign indicates *Vav-iCre*^*+*^, *Baf200*^*f/f*^ group mice started to die at 16 days post-transplantation. Data are shown as means ± SEM. **P < 0.01; ***P < 0.001

The aforementioned results showed that Baf200 plays important roles in normal hematopoiesis; we then asked whether Baf200 also has a role in leukemogenesis. To test this, we utilized a previously described acute myeloid leukemia (AML) mouse model induced by MLL-AF9 fusion protein (Fig. 7a) [43]. Leukemia cells were harvested from two cohorts of primary recipients, and 50,000, 200,000, or 500,000 cells were transplanted into sublethally irradiated recipients in the secondary transplantation. Notably, *Baf200* deficiency significantly accelerated the leukemia progression in the secondary transplantation. All mice in *Vav-iCre*^*+*^, *Baf200*^*f/f*^ group died at around 25 days post-transplantation, whereas the average survival lifespan of recipients in WT group behaved in a dose-dependent manner (Fig. 7b). We then adopted 200,000 donor cells as a standard in the follow-up experiments. *Vav-iCre*^*+*^, *Baf200*^*f/f*^ group mice showed much paler bones and splenomegaly at 20 days post-transplantation (Fig. 7c). Histology evaluation revealed more leukemia blasts in the peripheral blood, BM, spleen, and liver in *Vav-iCre*^*+*^, *Baf200*^*f/f*^ group recipients than WT group (Fig. 7d), and flow cytometry confirmed much more GFP^+^ leukemia cells in the peripheral blood, BM, and spleen from *Vav-iCre*^*+*^, *Baf200*^*f/f*^ group recipients (Fig. 7e). The *Vav-iCre*^*+*^, *Baf200*^*f/f*^ group mice consistently exhibited increased myeloid cells and decreased lymphoid cells in the peripheral blood compared with WT mice (Fig. 7f, g). In addition, *Vav-iCre*^*+*^, *Baf200*^*f/f*^ group mice showed significantly accelerated accumulation of leukemia cells in the peripheral blood (Fig. 7h).

**Fig. 8:**
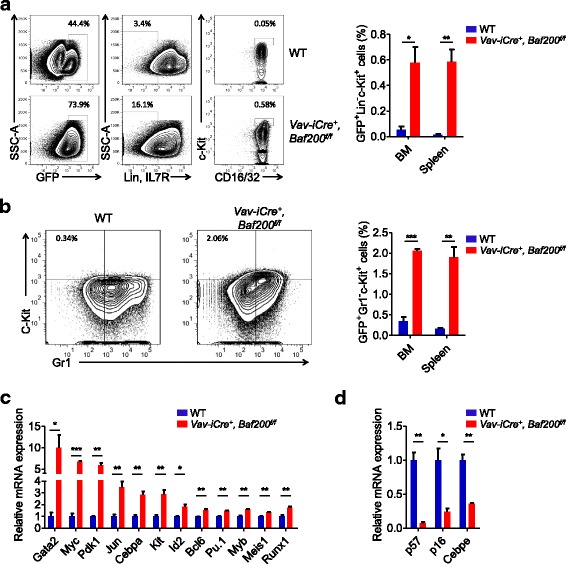
Increased percentage of LSCs in Baf200-deficient AML. **a** The flow profiles (left) and graph (right) showing the percentage of LSCs (GFP^+^Lin^−^c-Kit^+^) in the BM from recipients (*n* = 3 per genotype). **b** The flow profiles (left) and graph (right) showing the percentage of GFP^+^Gr1^−^c-Kit^+^ subset in the BM from recipients (*n* = 3 per genotype). **c**, **d** Upregulation (**c**) and downregulation (**d**) genes in *Vav-iCre*^+^, *Baf200*^*f/f*^ leukemia cells (*n* = 3 per genotype). Data are shown as means ± SEM. *P < 0.05; **P < 0.01; ***P < 0.001

We then detected the leukemia stem cell (LSC) frequency in WT and *Vav-iCre*^*+*^, *Baf200*^*f/f*^ recipients and found that the percentage of LSC-enriched population (GFP^+^Lin^−^c-Kit^+^) were much higher in the BM and spleen of *Vav-iCre*^*+*^, *Baf200*^*f/f*^ group (Fig. 8a). Another set of markers for enriched LSCs confirmed an increase in the percentage of GFP^+^Gr1^−^c-Kit^+^ cells in *Vav-iCre*^*+*^, *Baf200*^*f/f*^ group (Fig. 8b). To explore the underlying mechanisms by which *Baf200* deficiency accelerates the leukemia progression, we sorted GFP^+^ BM leukemia cells from *Vav-iCre*^*+*^, *Baf200*^*f/f*^ and WT group and detected the expression of hematopoiesis-related genes by RT-qPCR assay. Interestingly, several genes associated with hematopoietic malignancies, including *Gata2*, *Cebpa*, *Pdk1*, *Myb*, *Myc*, and *Meis1* [44–48], were upregulated in *Vav-iCre*^*+*^, *Baf200*^*f/f*^ AML cells (Fig. 8c). The expression of *Cebpe*, a key transcription factor in granulopoiesis [49], was decreased in *Vav-iCre*^*+*^, *Baf200*^*f/f*^ AML cells (Fig. 8d). Furthermore, loss of Baf200 promotes the progression of leukemogenesis partially by inhibiting the expression of *p57* and *p16* (Fig. 8d). Taken together, the results indicate that Baf200 acts as a tumor suppressor in MLL-AF9-induced AML.

## Discussion

Although ATP-dependent SWI/SNF complex have been demonstrated to play pivotal roles in normal or malignant hematopoiesis [13–21], little is known about the function of PBAF-specific subunit Baf200 in these processes. Here, we utilized several hematopoietic lineage-specific *Cre* mouse lines to investigate the role of Baf200 in fetal and adult hematopoiesis, as well as in leukemogenesis. Our current study clearly showed the cell-intrinsic requirement of *Baf200* in hematopoietic cells, as FL HSCs from *Tie2-Cre*^*+*^, *Baf200*^*f/f*^ or *Vav-iCre*^*+*^, *Baf200*^*f/f*^ embryos and BM HSCs from *Vav-iCre*^*+*^, *Baf200*^*f/f*^ mice exhibited impaired capacity of long-term hematopoietic reconstitution. Moreover, we utilized adoptive transfer strategy with inducible *Mx1-Cre* system and verified the cell-intrinsic requirement for Baf200 in the FL or adult BM HSC maintenance.

We noticed that *Tie2-Cre*^*+*^, *Baf200*^*f/f*^ mice displayed much more severe phenotypes than *Vav-iCre*^*+*^, *Baf200*^*f/f*^ mice. *Tie2-Cre*^*+*^, *Baf200*^*f/f*^ embryos died at embryonic stage with defective fetal erythropoiesis, whereas *Vav-iCre*^+^, *Baf200*^*f/f*^ mice were born at expected Mendelian ratio with an appearance indistinguishable from their littermate controls. *Tie2-Cre* is originally expressed in hemogenic endothelium as early as E6.5, which leads to efficient deletion in endothelial cells and their progeny (HSCs and all definitive blood cells) [31, 50]. In contrast, *Vav-iCre* begins to be expressed at E9.5 and the expression was greatly enhanced at E12.5; thus, it causes high deletion efficiency in hematopoietic lineages but low deletion efficiency in endothelial cells [42, 51]. So we speculated one possibility is that Baf200 in endothelial cells may also play a role in fetal hematopoiesis. Endothelial cell-specific *Cre* strain, such as *VE-Cadherin-CreERT2*, would be useful for dissecting the precise role of Baf200 in endothelial cells in the future. In mouse, there are two waves of fetal hematopoiesis, primitive, and definitive. Primitive hematopoiesis produces erythroid and myeloid cells in the YS blood island at around E7.5. Definitive hematopoietic cells (transient erythroid and myeloid progenitors) arise from hemogenic endothelium in the YS at around E8.25, and these cells can migrate into the FL to give rise to erythroid and myeloid progeny. Generation of de novo HSCs takes place in the AGM region at around E10.5, soon the HSCs migrate into the FL, expand, and differentiate [1, 52]. Previous study revealed that *Tie2-Cre* mediates recombination in almost all primitive and definitive hematopoietic cells [50], while only definitive hematopoietic cells that have already homed into FL can express recombinase in *Vav-iCre* strain [42]. Therefore, another possibility of the distinct phenotypes observed in *Tie2-Cre* and *Vav-iCre* strain is the different timing of Cre activity.

Several SWI/SNF complex subunits have been reported to play important roles in hematopoiesis. Brg1, a catalytic subunit of the BAF and PBAF complexes, is involved in both primitive and definitive erythropoiesis [13, 14]. Baf250a, a subunit of the BAF complex, controls the pool size of FL HSCs [20]. Baf53a, a subunit of both BAF and the PBAF complex, is required for HSC maintenance [18]. Baf180, another unique subunit of PBAF complex, is required for long-term reconstitution potential of HSCs [22]. Our current study provided additional evidence that Baf200, a PBAF specific subunit, plays a key role in hematopoiesis. Although Baf200 and Baf180 are both the PBAF-specific subunits, Baf200 seems to have a more important role than Baf180 in hematopoiesis. *Baf200*-deficient HSCs were poorly competitive in long-term hematopoietic reconstitution following primary transplantation, whereas *Baf180*-deficient HSCs had a compromised long-term reconstitution potential only in secondary transplantation. BAF200 interacts with BAF180 and some other identical subunits within PBAF complex to exert their function [12]. However, a previous study showed that BAF200, but not BAF180, is essential for the stability of PBAF complex [53]. This may be an explanation for the appeared more vital role of Baf200 in hematopoiesis. However, whether Baf200 regulates hematopoiesis through a PBAF-independent mechanism needs to be determined in future studies.

Epigenetic modifiers may have adverse effects on normal and malignant hematopoiesis. For example, Asxl1, a polycomb group protein, is essential for preserving hematopoietic reconstitution capacity, whereas haploinsufficiency of *Asxl1* can cause MDS-like disease [54, 55]. Utx, a di- and trimethyl H3K27 demethylase, is required for normal hematopoiesis [56]; meanwhile, Utx serves as a tumor suppressor in NOTCH1-induced T cell acute lymphoblastic leukemia (T-ALL) [57]. Moreover, differentially expressed genes regulated by Utx were found in normal *Utx*-deficient BM cells or in *Utx*-deficient leukemia cells [56, 57], indicating Utx may regulate the target genes in a dual context-dependent manner. Our current study found that *Baf200* deficiency impairs normal hematopoiesis but accelerates MLL-AF9-driven leukemogenesis, suggesting that *Baf200* may play contrast roles in normal and malignant hematopoiesis. We also revealed diverse genes regulated by Baf200 in normal LSK cells and AML cells. However, whether *Baf200* regulate its target genes or pathways in a dual context-dependent manner in normal or leukemia cells still need further investigation.

Several potential *Baf200* targets were identified by RNA-seq analysis and RT-qPCR in our study. RNA-seq results revealed diverse patterns of gene differentially regulated by Baf200 in different cell lineages, including FL LSK, FL erythrocytes, adult BM LSK, and leukemia cells. These results indicate that Baf200 may regulate different subsets of target genes in a cell context-dependent manner. It may recruit different transcription factors or histone modifiers or be recruited by them to the specific target genes in different cell types. Whether Baf200 controls these genes through a direct or indirect manner still needs further investigations. Future studies will aim to explore the underlying mechanisms of Baf200 in normal and malignant hematopoiesis by using ChIP-based analysis.

## Conclusions

In summary, this study reveals that loss of Baf200 impairs the normal fetal and adult hematopoiesis while accelerates the progression of MLL-AF9-induced leukemia, which provide a potential therapeutic target for leukemia treatment.

## Additional file

Additional file 1: Table S1. The immunophenotypes of the tested subsets in Figure S1. Table S2. List of primers used in mice genotyping. Table S3. List of primers used in RT-qPCR experiments. Table S4. List of commercial available antibodies used in these studies. Figure S1. Expression pattern of the *Baf200* gene in FACS-purified populations from mouse FL, BM, and spleen (*n* = 3). Figure S2. Baf200 is dispensable for the proliferation and apoptosis of FL erythrocytes. Figure S3. Gene Ontology analysis of *Baf200*-regulated genes in FL S3 cells. Figure S4. RNA-seq analysis of FL LSK cells from WT and *Tie2-Cre*^*+*^, *Baf200*^*f/f*^ embryos. Figure S5. FL HSCs from *Tie2-Cre*^*+*^, *Baf200*^*f/f*^ embryos show impaired long-term reconstitution potential, related to Fig. 3. Figure S6. Baf200 contributes to adult erythropoiesis and hematopoiesis, related to Figs. 4 and 5. Figure S7. Baf200 is dispensable for the cell cycle status or apoptosis of BM LSK compartment in steady state. Figure S8. Gene Ontology analysis of *Baf200*-regulated genes in BM LSK cells. Figure S9. Cell-intrinsic role of Baf200 in HSCs function, related to Fig. 6. Figure S10. FL HSCs from *Vav-iCre*^*+*^, *Baf200*^*f/f*^ mice show impaired long-term reconstitution potential. (DOCX 1524 kb)

## Abbreviations

AGM: Aorta-gonad-mesonephros
AML: Acute myeloid leukemia
ATP: Adenosine triphosphate
BAF: BRG1-associated factor
BM: Bone marrow
CMP: Common myeloid progenitor
FL: Fetal liver
GMP: Granulocyte-macrophage progenitor
GO: Gene Ontology
HSC: Hematopoietic stem cell
HSPCs: Hematopoietic stem and progenitor cells
LSC: Leukemic stem cell
LSK: Lineage^−^Sca1^+^c-Kit^+^
LT-HSC: Long-term hematopoietic stem cell
MDS: Myelodysplastic syndrome
MEP: Megakaryocyte–erythroid progenitor
MPP: Multipotent progenitor
PBAF: Polybromo BRG1-associated factor
ProE: Pro-erythroblast
RT-qPCR: Reverse transcription-quantitative polymerase chain reaction
ST-HSC: Short-term hematopoietic stem cell
WT: Wild-type
YS: Yolk sac
