# Supplementary material for: The chromatin remodeling subunit Baf200 promotes normal hematopoiesis and inhibits leukemogenesis
Source: J Hematol Oncol. 2018 Feb 26;11:27. doi: 10.1186/s13045-018-0567-7 (PMC5828314; doi:10.1186/s13045-018-0567-7)
Supplement: Supplementary file 1 — The immunophenotypes of the tested subsets in Figure S1. Table S2. List of primers used in mice genotyping. Table S3. List of primers used in RT-qPCR experiments. Table S4. List of commercial available antibodies used in these studies. Figure S1. Expression pattern of the Baf200 gene in FACS-purified populations from mouse FL, BM, and spleen (n = 3). Figure S2. Baf200 is dispensable for the proliferation and apoptosis of FL erythrocytes. Figure S3. Gene Ontology analysis of Baf200-regulated genes in FL S3 cells. Figure S4. RNA-seq analysis of FL LSK cells from WT and Tie2-Cre+, Baf200f/f embryos. Figure S5. FL HSCs from Tie2-Cre+, Baf200f/f embryos show impaired long-term reconstitution potential, related to Fig. 3. Figure S6. Baf200 contributes to adult erythropoiesis and hematopoiesis, related to Figs. 4 and 5. Figure S7. Baf200 is dispensable for the cell cycle status or apoptosis of BM LSK compartment in steady state. Figure S8. Gene Ontology analysis of Baf200-regulated genes in BM LSK cells. Figure S9. Cell-intrinsic role of Baf200 in HSCs function, related to Fig. 6. Figure S10. FL HSCs from Vav-iCre+, Baf200f/f mice show impaired long-term reconstitution potential. (DOCX 1524 kb) [file 13045_2018_567_MOESM1_ESM.docx]

**Additional file1**

**The chromatin remodeling subunit Baf200 promotes normal hematopoiesis and inhibits leukemogenesis in mice**

Lulu Liu^1,2^, Xiaoling Wan^2,3^, Peipei Zhou^2,3^, Xiaoyuan Zhou^3,5^, Wei Zhang^2,7^, Xinhui Hui^2,7^, Xiujie Yuan^2,3^, Xiaodan Ding^2,3^, Ruihong Zhu^2,3^, Guangxun Meng^2,3^, Hui Xiao^2,3^, Feng Ma^6^, He Huang^8^, Xianmin Song^9^, Bin Zhou^4,*^, Sidong Xiong^1,*^ and Yan Zhang^2,3,*^

^1^Institute of Biology and Medical Sciences, Soochow University, Suzhou, China; ^2^Key Laboratory of Molecular Virology and Immunology, Institut Pasteur of Shanghai, Chinese Academy of Sciences, Shanghai, China; ^3^University of Chinese Academy of Sciences, Beijing, China; ^4^The State Key Laboratory of Cell Biology, CAS Center for Excellence in Molecular Cell Science, Shanghai Institute of Biochemistry and Cell Biology, Chinese Academy of Sciences, Shanghai, China; ^5^CAS-MPG Partner Institute for Computational Biology, Shanghai Institutes for Biological Sciences, Chinese Academy of Sciences, Shanghai, China; ^6^Institute of Blood Transfusion, Chinese Academy of Medical Sciences and Peking Union Medical College, Chengdu, China; ^7^Shcool of Life Sciences, Shanghai University, Shanghai, China; ^8^Bone Marrow Transplantation Center, the First Affiliated Hospital, Zhejiang University School of Medicine, Hangzhou, China; ^9^Department of Hematology, Shanghai Jiao Tong University Affiliated Shanghai General Hospital, Shanghai, China.

**Corresponding authors**:

Bin Zhou

Shanghai Institute of Biochemistry and Cell Biology, Chinese Academy of Sciences

320 Yue-Yang Road, Shanghai, China

Email: [zhoubin@sibs.ac.cn](mailto:zhoubin@sibs.ac.cn)

Phone/Fax: (+86)-21-54920974

Sidong Xiong

Institute of Biology and Medical Sciences (IBMS), Soochow University

No. 199 Ren'ai Rd, Suzhou, China

Email: [sdxiong@suda.edu.cn](mailto:sdxiong@suda.edu.cn)

Phone/Fax: (+86)-512-65882135

Yan Zhang

Institut Pasteur of Shanghai, Chinese Academy of Sciences

320 Yue-Yang Road, Shanghai, China

Email: [yan_zhang@sibs.ac.cn](mailto:yan_zhang@sibs.ac.cn)

Phone/Fax: (+86)-21-54923137

**Table S1.** The immunophenotypes of the tested subsets in supplemental Figure 1.

| LT-HSC | Lin^-^Sca1^+^c-Kit^+^CD34^-^Flt3^low^ |
| --- | --- |
| ST-HSC | Lin^-^Sca1^+^c-Kit^+^CD34^+^Flt3^low^ |
| MPP | Lin^-^Sca1^+^c-Kit^+^CD34^+^Flt3^+^ |
| LSK | Lin^-^Sca1^+^c-Kit^+^ |
| HPC | Lin^-^Sca1^-^c-Kit^+^ |
| CMP | Lin^-^Sca1^-^c-Kit^+^CD34^+^CD16/32^low^ |
| CLP | Lin^-^IL7R^+^Sca1^low^c-Kit^low^ |
| MEP | Lin^-^Sca1^-^c-Kit^+^CD34^-^CD16/32^low^ |
| GMP | Lin^-^Sca1^-^c-Kit^+^CD34^+^CD16/32^high^ |
| S0+S1 | Ter119^-^CD71^+/-^ |
| S2 | Ter119^low^CD71^+^ |
| S3 | Ter119^high^CD71^+^ |

**Table S2.** List of primers used in mice genotyping.

|  | Genotyping primers |
| --- | --- |
| *Baf200 ^f/f^* mice | Pr1794:TCCTACAACCTGGCCTTATGGAGG  Pr1795:AGCAAAGTACAAGTGCCTGTTGG  Pr1796:GCAAATAAAGCCTTAAGCAC |
| *Vav-iCre* transgenic mice | Vav-F:AGATGCCAGGACATCAGGAACCTG  Vav-R:ATCAGCCACACCAGACACAGAGATC |
| *Tie2-Cre* transgenic mice | Tie2-F:CGCATAACCAGTGAAACAGCATTGC  Tie2-F:CCCTGTGCTCAGACAGAAATGAGA |
| *Mx1-Cre* transgenic mice | Mx1-F:CGGTCGATGCAACGAGTGATGAGG  Mx1-R:CCAGAGACGGAAATCCATCGTCG |

**Table S3.** List of primers used in RT-qPCR experiments.

| Gene | Sequence (5’-3’) |
| --- | --- |
| *Baf200* | Pr2563:AGAAGTTGTTCCAACGCTGCC  Pr2564: TGTGGCTTTGGATTGCCTGG |
| *Bnip3* | F: TCCTGGGTAGAACTGCACTTC  R: GCTGGGCATCCAACAGTATTT |
| *Csf1r* | F: TGTCATCGAGCCTAGTGGC  R: CGGGAGATTCAGGGTCCAAG |
| *Hmox1* | F: AAGCCGAGAATGCTGAGTTCA  R:GCCGTGTAGATATGGTACAAGGA |
| *Ptpn6* | F: GGACTTCTATGACCTGTACGGA  R:CGAGCAGTTCAGTGGGTACTT |
| *Sap30l* | F: CAGAAAAGCATCTCGCAGAAGA  R: CCATCGTCACTTGCCTTCCTC |
| *Gpi1* | F: TCAAGCTGCGCGAACTTTTTG  R: GGTTCTTGGAGTAGTCCACCAG |
| *Csf2rb* | F: GTGGAGCGAAGAGTACACTTG  R: CCAAAGCGAAGGATCAGGAG |
| *Hmga1* | F: GGTCGGGAGTCAGAAAGAGC  R: ATTCTTGCTTCCCTTTGGTCG |
| *Myb* | F: ATCAAGCAGGAGGTGGAGTC  R: CGGTAAAGGCTTTGAGGACA |
| Stat1 | F: CGGAGTCGGAGGCCCTAAT  R: ACAGCAGGTGCTTCTTAATGAG |
| *Kit* | F: AGGCTATCCCTGTTGTGTCTG  R: ACATGGAGTTCACGGATGTAGA |
| *Hbb-y* | F: TGGCCTGTGGAGTAAGGTCAA  R: GAAGCAGAGGACAAGTTCCCA |
| *Fbxo6* | F: TCCCTATGGAAGCGCAAGAGT  R: CTCCGTTGGAGTCTATCCGC |
| *Zfp78* | F: TCTGGGCAGGGAAGACCATT  R: CCGCAGACGGTGCATTGATA |
| *Cd36* | F: ATGGGCTGTGATCGGAACTG  R: GTCTTCCCAATAAGCATGTCTCC |
| Pla2g16 | F: GACGAGGAGTACACCCCACT  R: CTCACAGTTCTCGCTGGTCA |
| *Gapdh* | F: CATGTTCGTCATGGGTGTGAACCA  R: AGTGATGGCATGGACTGTGGTCAT |
| *Rnasel* | F: TAGGCGAACACATCAATGAGGA  R: CTGCCTCTGGAACGCTGAG |
| *Rbl2* | F: TCCTTACACGACGGTCTAGTG  R: TCCCAGCGGGTAACACGTA |
| *Procr* | F: AATGCCTACAACCGGACTCG  R: ACCAGTGATGTGTAAGAGCGA |
| *Fstl1* | F: CACGGCGAGGAGGAACCTA  R: TCTTGCCATTACTGCCACACA |
| *Gprasp2* | F: ATGGGTTCTTGGTGCTATCCC  R: CCTGGACCTAGTGTTCACCTC |
| *Tbxas1* | F: TACCATAGTGACTGTGACTCTGC  R: GGTGCCTGATGCCCAACTT |
| *Hck* | F: GGCATGGCCTTCATTGAGC  R: CAGCAATCTTACACACCAGTGA |
| *Add2* | F: CTGACACTGATGGCGACAG  R: AACGAGGGGGTTCGGAATTTC |
| *Cldn13* | F: ATGGTCGTCAGCAAACAAGAG  R: CATCATCTGGAAAGGTCACCC |
| *Vpreb1* | F: GCTGCTGGCCTATCTCACAG  R: CCAATGTTATGGTCGTTGCTCA |
| *Pou2af1* | F: CACCAAGGCCATACCAGGG  R: GAAGCAGAAACCTCCATGTCA |
| *Bex* | F: ATGGAGTCCAAAGATCAAGGCG  R: CTGGCTCCCTTCTGATGGTA |
| *Fut10* | F: CTGGGCGCTTTTCCATGAAGA  R: TAGTCAGTGGCAAGTGGGAAT |
| *Rhou* | F: GGCTACCCCACCGAGTACAT  R: GGGGCCTCAGCTTGTCAAA |
| *Blk* | F: GAGGCAGGTCAGTGAGAAGG  R: GTCCTGGTTAGGAGATGGTGG |
| *Gdf3* | F: ATGCAGCCTTATCAACGGCTT  R: AGGCGCTTTCTCTAATCCCAG |
| *Mllt4* | F: AAGCTGGCCGACATCATTCAC  R: GCTGTGCTAGAGACTCGAATACA |
| *Glis2* | F: GACGAGCCCCTCGACCTAA  R: AGCTCTCGATGCAAAGCATGA |
| *Icos* | F: ATGAAGCCGTACTTCTGCCAT  R: CGCATTTTTAACTGCTGGACAG |
| *Fcer1a* | F: GAGTGCCACCGTTCAAGACA  R: GTAGATCACCTTGCGGACATTC |
| *Prdm5* | F: GAGCTTTTGATTGGCTACCTGG  R: GTCCCTTAGAGTGGTCAACTTTG |
| *p27* | F: TCAAACGTGAGAGTGTCTAACG  R: CCGGGCCGAAGAGATTTCTG |
| *p16* | F: CGCAGGTTCTTGGTCACTGT  R: TGTTCACGAAAGCCAGAGCG |
| *Lats2* | F: GGACCCCAGGAATGAGCAG  R: CCCTCGTAGTTTGCACCACC |
| *Cebpa* | F: CAAGAACAGCAACGAGTACCG  R: GTCACTGGTCAACTCCAGCAC |
| *Bcl6* | F: CCGGCACGCTAGTGATGTT  R: TGTCTTATGGGCTCTAAACTGCT |
| *Myc* | F: CCCTATTTCATCTGCGACGAG  R: GAGAAGGACGTAGCGACCG |
| *Meis1* | F: TCAGCAAATCTAACTGACCAGC  R: AGCTACACTGTTGTCCAAGCC |
| *Pdk1* | F: GGACTTCGGGTCAGTGAATGC  R: TCCTGAGAAGATTGTCGGGGA |
| *Cebpe* | F: ATTCGCCTATCCCTCACACAC  R: GTAGCTGCCTCGACTGGTG |
| *Gata2* | F: CACCCCGCCGTATTGAATG  R: CCTGCGAGTCGAGATGGTTG |
| *Jun* | F: CCTTCTACGACGATGCCCTC  R: GGTTCAAGGTCATGCTCTGTTT |
| *Id2* | F: ATGAAAGCCTTCAGTCCGGTG  R: AGCAGACTCATCGGGTCGT |
| *Pu.1* | F: ATGTTACAGGCGTGCAAAATGG  R: TGATCGCTATGGCTTTCTCCA |
| *Runx1* | F: GCAGGCAACGATGAAAACTACT  R: GCAACTTGTGGCGGATTTGTA |
| *p57* | F: CGAGCAGAGCAGGACGAATC   \| R: GAAGAAGTCGTTCGCATTGGC \|  \| \| --- \| --- \| |

**Table S4.** List of commercial available antibodies used in these studies**.**

| Antibodies | Cat. No. | Company |
| --- | --- | --- |
| Anti-Mouse-CD11b biotin | 13-0112 | eBioscience |
| Anti-Mouse-CD3e biotin | 13-0031 | eBioscience |
| Anti-Mouse-CD45R biotin | 13-0452 | eBioscience |
| Anti-Mouse-Ter119 biotin | 13-5921 | eBioscience |
| Anti-Mouse-CD8 biotin | 13-0081 | eBioscience |
| Anti-Mouse-CD4 biotin | 13-0042 | eBioscience |
| Anti-Mouse-CD5 biotin | 13-0051 | eBioscience |
| Anti-Mouse-Gr-1 biotin | 13-5931 | eBioscience |
| Anti-Mouse-CD41 biotin | 13-0411 | eBioscience |
| Anti-Mouse-Sca1 FITC | 11-5981 | eBioscience |
| Anti-Mouse-CD45.1 FITC | 11-0453 | eBioscience |
| Anti-Mouse-CD4 FITC | 11-0042 | eBioscience |
| Anti-Mouse-CD71 FITC | 11-0711 | eBioscience |
| Anti-Mouse-CD11b FITC | 11-0112 | eBioscience |
| Anti-Mouse-CD34 FITC | 11-0341 | eBioscience |
| Anti-Mouse-CD48 FITC | 11-0481 | eBioscience |
| Anti-Mouse-Ki67 FITC | 11-5698 | eBioscience |
| Anti-Mouse-IgM FITC | 11-5890 | eBioscience |
| Anti-Mouse-CD4 FITC | 11-0042 | eBioscience |
| Anti-Mouse-MHCⅡ FITC | 11-5321 | eBioscience |
| Streptavidin FITC | 11-4317 | eBioscience |
| Anti-Mouse-CD45.2 PE | 12-0454 | eBioscience |
| Anti-Mouse-Ter119 PE | 12-5921 | eBioscience |
| Anti-Mouse- CD117 (c-Kit) PE | 12-1172 | eBioscience |
| Anti-Mouse-CD150 PE | 12-1502 | eBioscience |
| Anti-Mouse-CD44 PE | 12-0441 | eBioscience |
| Anti-Mouse-CD11c PE | 12-0114 | eBioscience |
| Anti-Mouse-CD127 PE | 12-1271 | eBioscience |
| Anti-Mouse-CD45R PE | 12-0452 | eBioscience |
| Anti-Mouse-CD8a PE | 12-0081 | eBioscience |
| Anti-Mouse-CD135 PE | 12-1351 | eBioscience |
| Anti-Mouse-CD3e PE | 12-0031 | eBioscience |
| Anti-Mouse-CD16/32 PE | 12-0161 | eBioscience |
| Anti-Mouse-Gr-1 PE | 12-5931 | eBioscience |
| Anti-Mouse-Ter119 PE | 12-5921 | eBioscience |
| Anti-Mouse CD71 APC | 17-0711 | eBioscience |
| Anti-Mouse-Sca1 APC | 17-5981 | eBioscience |
| Anti-Mouse CD19 APC | 17-0193 | eBioscience |
| Anti-Mouse CD3e APC | 17-0031 | eBioscience |
| Anti-Mouse CD11b APC | 17-0112 | eBioscience |
| Anti-Mouse Ly-6G (Gr-1) APC | 17-5931 | eBioscience |
| Anti-Mouse CD45.2 APC-eFluor® 780 | 47-0454 | eBioscience |
| [Anti-Mouse CD117 (c-Kit) APC-eFluor® 780](http://www.ebioscience.com/mouse-cd117-c-kit-antibody-apc-efluor-780-ack2.htm) | 47-1172 | eBioscience |
| Anti-Mouse CD8a APC-eFluor® 780 | 47-0081 | eBioscience |
| Streptavidin PE-Cyanine7 | 25-4317 | eBioscience |
| Anti-Mouse Ki67 PE-Cyanine7 | 25-5698 | eBioscience |
| Anti-Mouse CD4 PE-Cyanine7 | 25-0041 | eBioscience |
| Anti-Mouse CD127 PE-Cyanine7 | 25-1271 | eBioscience |
| Anti-Mouse CD45.1 BV421 | 563983 | BD Biosciences |
| Anti-Mouse CD45.2 BV786 | 563686 | BD Biosciences |


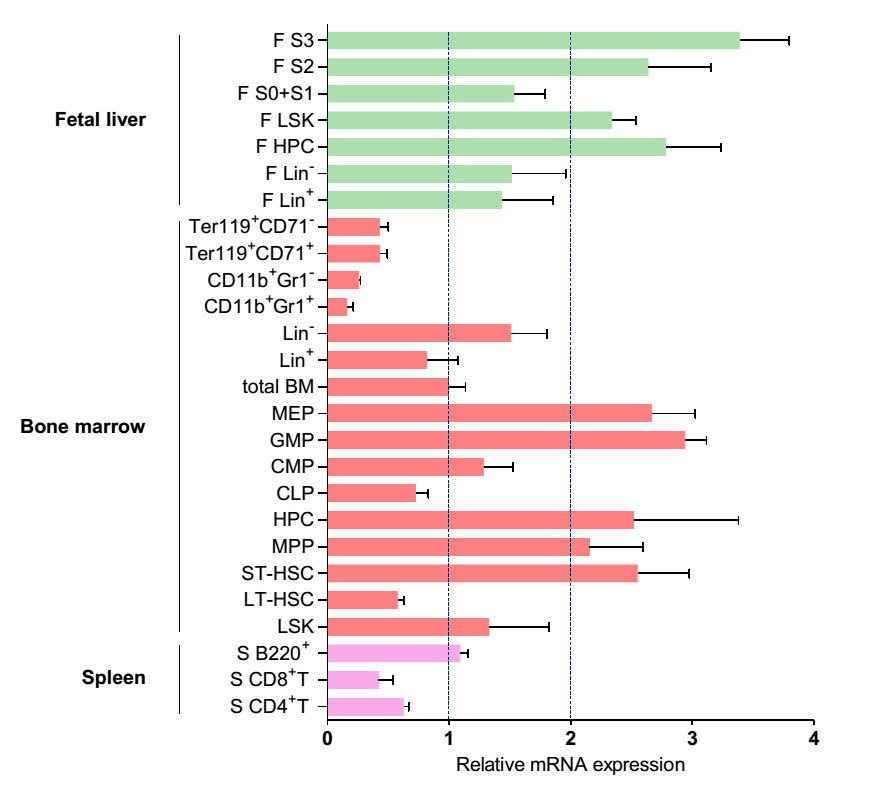


**Figure S1.** Expression pattern of the *Baf200* gene in FACS-purified populations from mouse FL, BM, and spleen (n=3). Total RNA isolated from diverse cell types was reverse-transcribed and analyzed by quantitative PCR. mRNA levels were normalized to the *Gapdh* expression. “F”, BM, and “S” indicate fetal liver, bone marrow and spleen, respectively. LT-HSC: Long-term hematopoietic stem cell; ST-HSC: Short-term hematopoietic stem cell; LSK: lineage^-^Sca1^+^ c-Kit^+^; HPC: hematopoietic progenitor cell; MPP: Multipotent progenitor; CMP: Common myeloid progenitor; MEP: Megakaryocyte-erythroid progenitor; GMP: Granulocyte-macrophage progenitor; CLP: Common lymphoid progenitor. The expression of *Baf200* in total BM cells is regarded as 1.000. Data are shown as means±SEM. See Additional file 1: Table S1 for the immunophenotypes of the different populations tested.


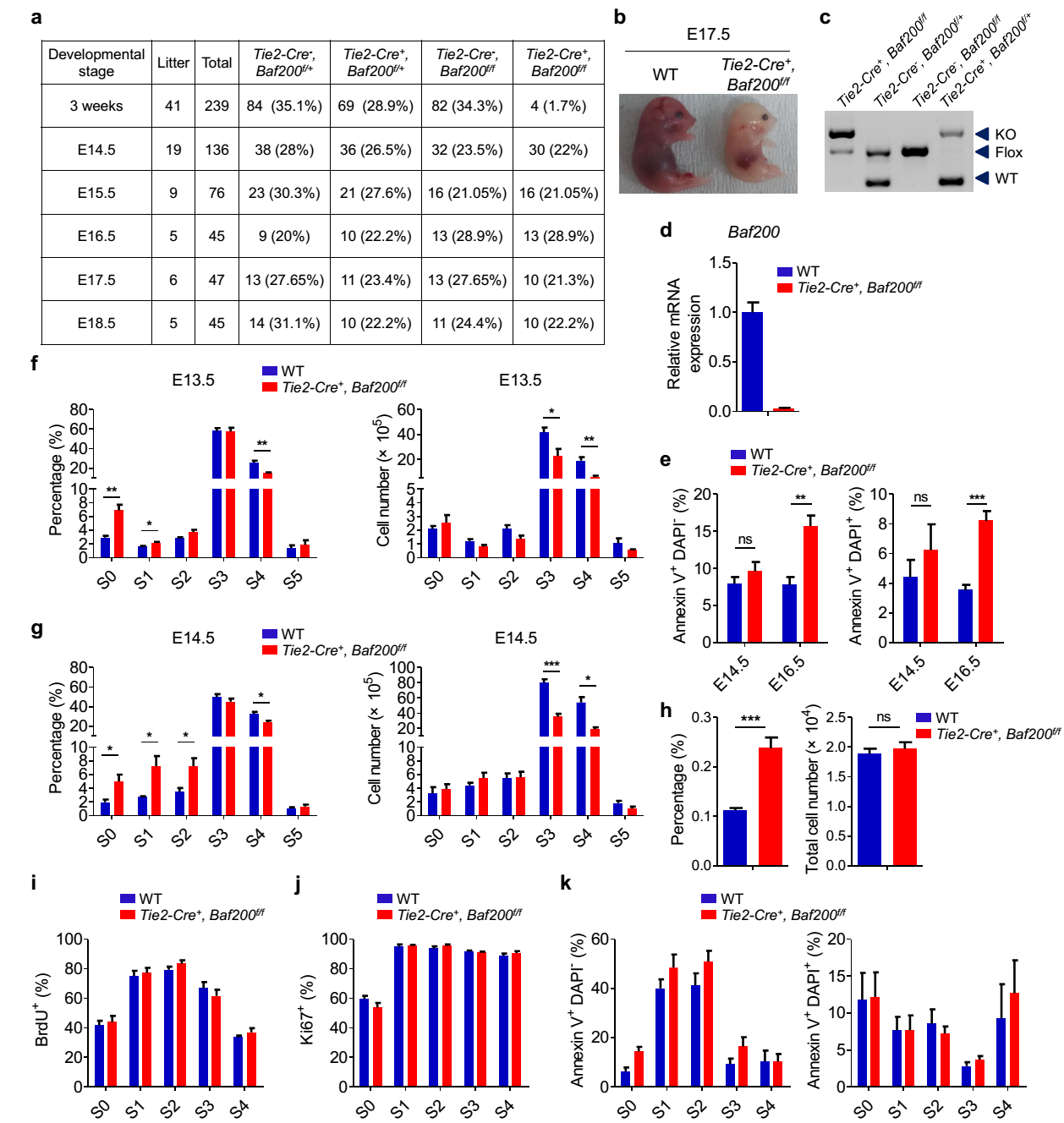


**Figure S2.** Baf200 is dispensable for the proliferation and apoptosis of FL erythrocytes. **(a)** The offspring from intercross between male *Tie2-Cre^+^, Baf200^f/+^* mice and female *Tie2-Cre^-^, Baf200^f/f^* mice at indicated stages. **(b)** Representative photograph of E17.5 *Tie2-Cre^+^, Baf200^f/f^* embryos and WT littermate. **(c-d)** Deletion efficiency of *Baf200* in *Tie2-Cre^+^, Baf200^f/f^* FL cells was confirmed by genomic PCR (**c**) or RT-qPCR (**d**). “WT,” “Flox,” and “KO” alleles indicate the wild-type *Baf200* allele, floxed *Baf200* allele, and exon4 deleted *Baf200* allele, respectively. **(e)** Graph showing the apoptosis status of *Tie2-Cre^+^, Baf200^f/f^* and WT FL cells at E14.5 and E16.5 (n=4 per genotype for each subset). **(f)** Percentage (left) and absolute cell number (right) of CD71/Ter119 erythroid subsets in E13.5 *Tie2-Cre^+^, Baf200^f/f^* embryos and WT littermates (n=4 per genotype for each subset). **(g)** Percentage (left) and absolute cell number (right) of CD71/Ter119 erythroid subsets in E14.5 *Tie2-Cre^+^, Baf200^f/f^* embryos and WT littermates (n=3-4 per genotype for each subset). **(h)** Percentage (left) and absolute cell number (right) of MEP in E14.5 WT and *Tie2-Cre^+^, Baf200^f/f^* FLs. **(i-j)** Graph showing the percentage of BrdU^+^ cells (**i**) (n=8 per genotype for each subset) and Ki67^+^ cells (**j**) (n=5 per genotype for each subset) in the indicated cell types from E13.5 *Tie2-Cre^+^, Baf200^f/f^* embryos and WT littermates. **(k)** Graph showing the apoptosis status of the indicated cell types from E13.5 *Tie2-Cre^+^, Baf200^f/f^* embryos and WT littermates (n=5 per genotype for each subset). Data are shown as means±SEM. *P<0.05; **P<0.01; ***P<0.001.


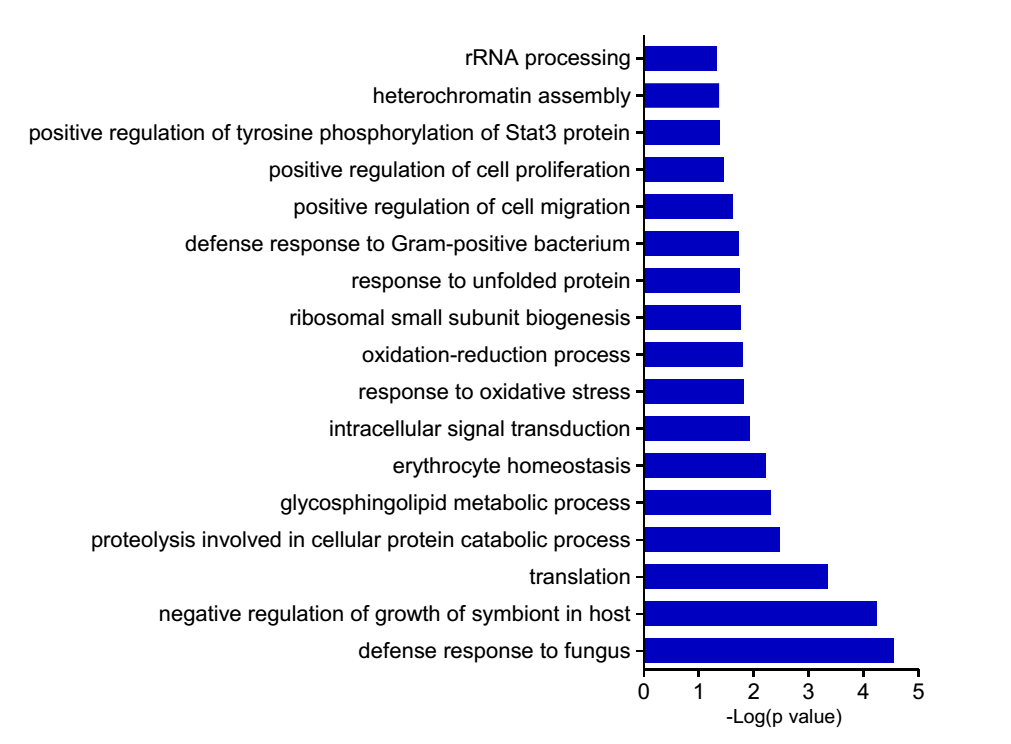


**Figure S3.** Gene Ontology analysis of *Baf200*-regulated genes in FL S3 cells. Transcripts showing over 2-fold difference in expression were used in the analysis and results are expressed as –log (p value).


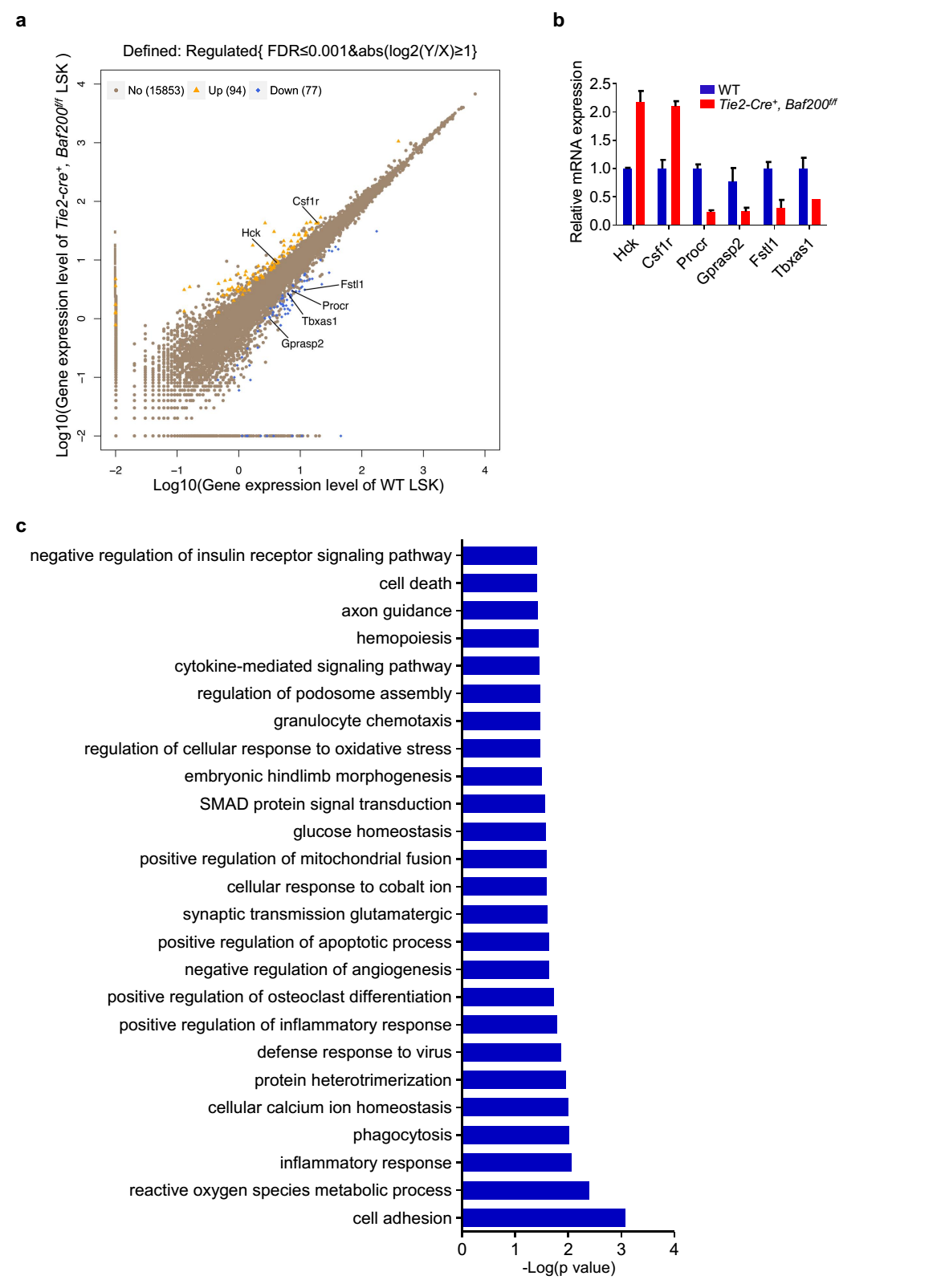


**Figure S4.** RNA-seq analysis of FL LSK cells from WT and *Tie2-Cre^+^, Baf200^f/f^* embryos. **(a)** Scatter plots of all expressed genes in LSK cells from WT and *Tie2-Cre^+^, Baf200^f/f^* FLs. Blue indicates down-regulation gene, yellow indicates up-regulation gene, and grey indicates non-regulation gene in *Tie2-Cre^+^, Baf200^f/f^* LSK cells. **(b)** Up-regulation genes and down-regulation genes in *Tie2-Cre^+^, Baf200^f/f^* LSK cells were confirmed by RT-qPCR (n=2 per genotype). Data are shown as means±SEM. **(c)** Gene Ontology analysis of *Baf200*-regulated genes in FL LSK cells. Transcripts showing over 2- fold difference in expression were used in the analysis and results are expressed as –log (p value).


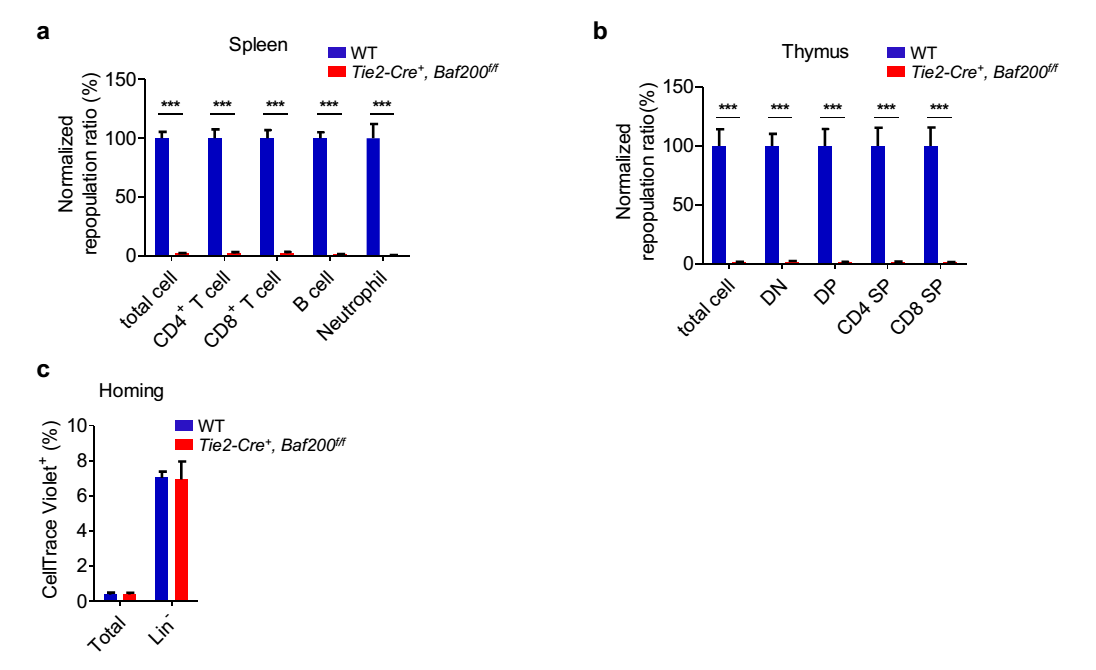


**Figure S5.** FL HSCs from *Tie2-Cre^+^, Baf200^f/f^* embryos show impaired long-term reconstitution potential, related to Figure 3. **(a-b)** The graph showing the relative ratios of CD45.2 versus CD45.1 of the indicated cell types in the spleen (**a**) and thymus (**b**) from recipient mice 16 weeks after transplantation (n=6 recipient mice per donor genotype). **(c)** Graph showing the percentage of donor cells in homing assay (n=3 recipient mice per donor genotype). Data are shown as means±SEM. ***P<0.001.


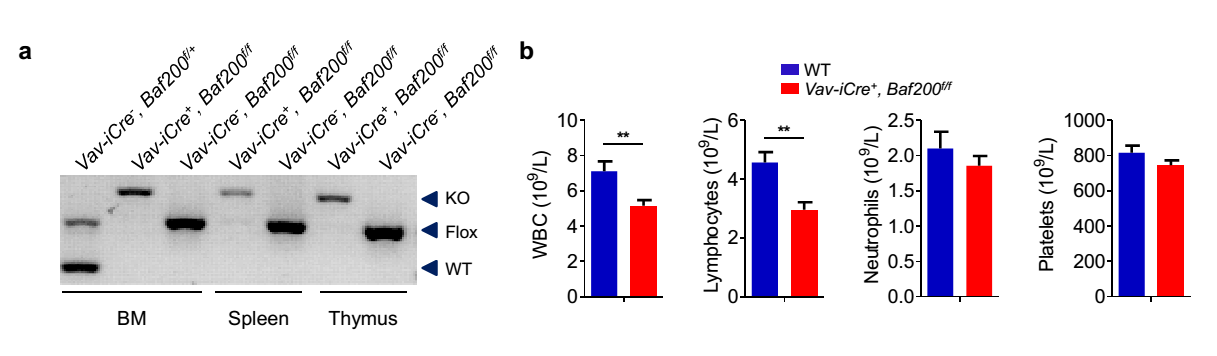


**Figure S6.** Baf200 contributes to adult erythropoiesis and hematopoiesis, related to Figure 4 and Figure 5. **(a)** Deletion efficiency of *Baf200* in *Vav-iCre^+^, Baf200^f/f^* BM, spleen, and thymus was confirmed by genomic PCR. “WT,” “Flox,” and “KO” alleles indicate the wild-type *Baf200* allele, floxed *Baf200* allele, and exon4 deleted *Baf200* allele, respectively. **(b)** Graph showing the number of WBC (white blood cell), lymphocytes, neutrophils, and platelets in the peripheral blood from WT (n=8 per genotype) and *Vav-iCre^+^, Baf200^f/f^* mice (n=9 per genotype). Data are shown as means±SEM. **P<0.01.


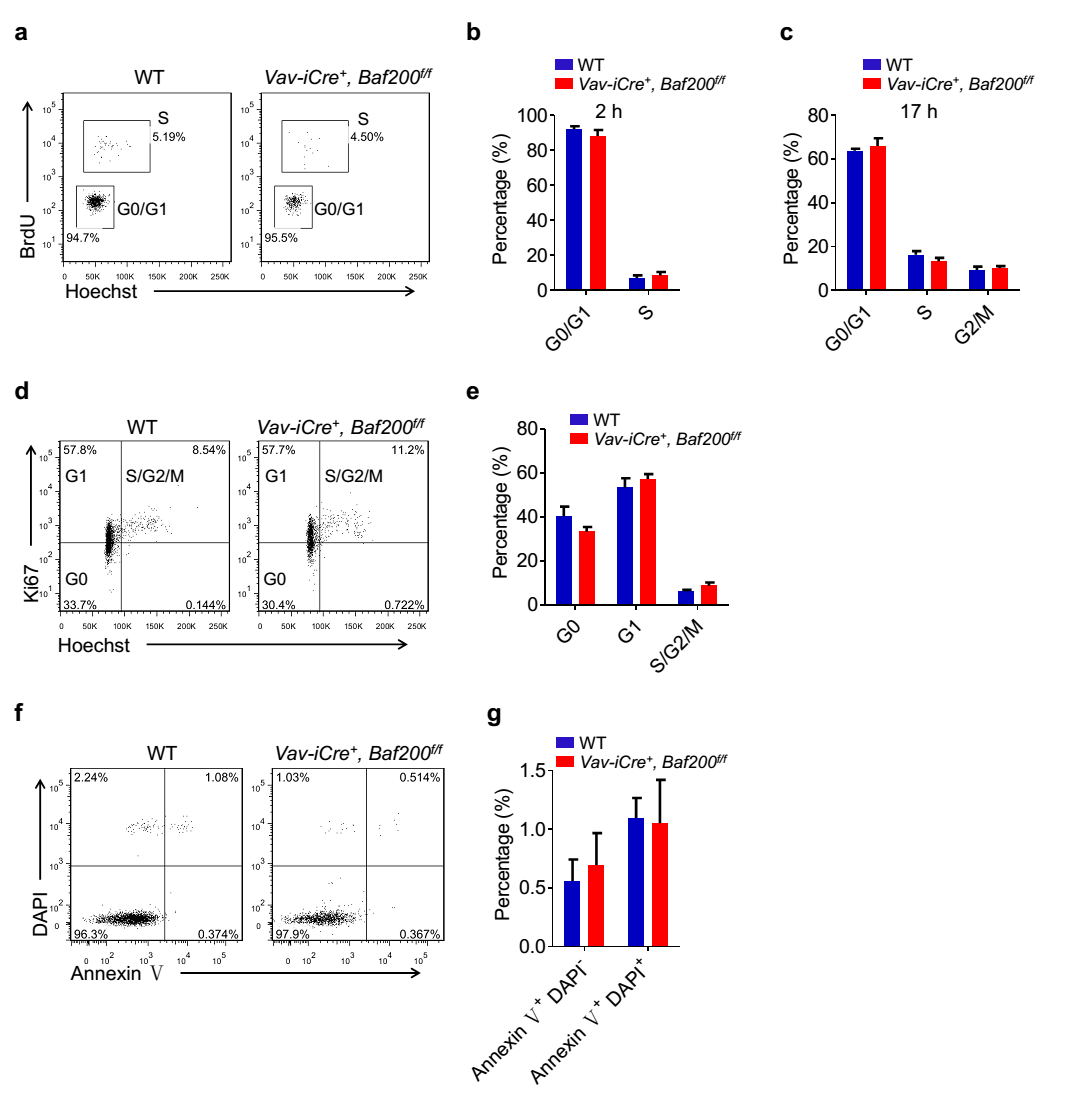


**Figure S7.** Baf200 is dispensable for the cell-cycle status or apoptosis of BM LSK compartment in steady-state. **(a-e)** Cell cycle status of LSK cells in the BM from WT and *Vav-iCre^+^, Baf200^f/f^* mice. **(a)** FACS profiles of BrdU incorporation assay. **(b-c)** Graph showing the results of BrdU incorporation assay (BrdU treatment for 2 hours in **c** or 17 hours in **d**) (n=4-5 per genotype). **(d)** FACS profiles of Ki67 staining assay. **(e)** Graph showing the result of Ki67 staining assay (n=6 per genotype). **(f-g)** FACS profiles (**f**) and graph (**g**) showing the apoptosis status of BM LSK cells from WT and *Vav-iCre^+^, Baf200^f/f^* mice (n=7 per genotype). Data are shown as means±SEM.


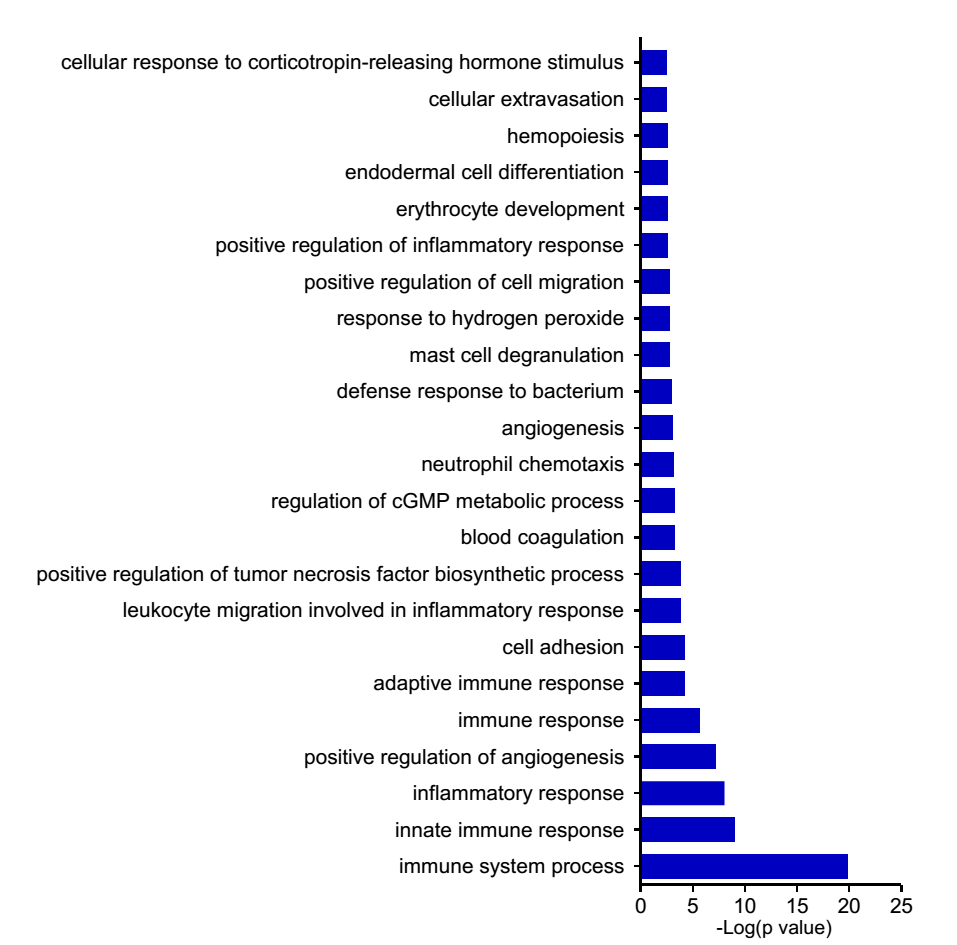


**Figure S8.** Gene Ontology analysis of *Baf200*-regulated genes in BM LSK cells. Transcripts showing over 2-fold difference in expression were used in the analysis and results are expressed as –log (p value).


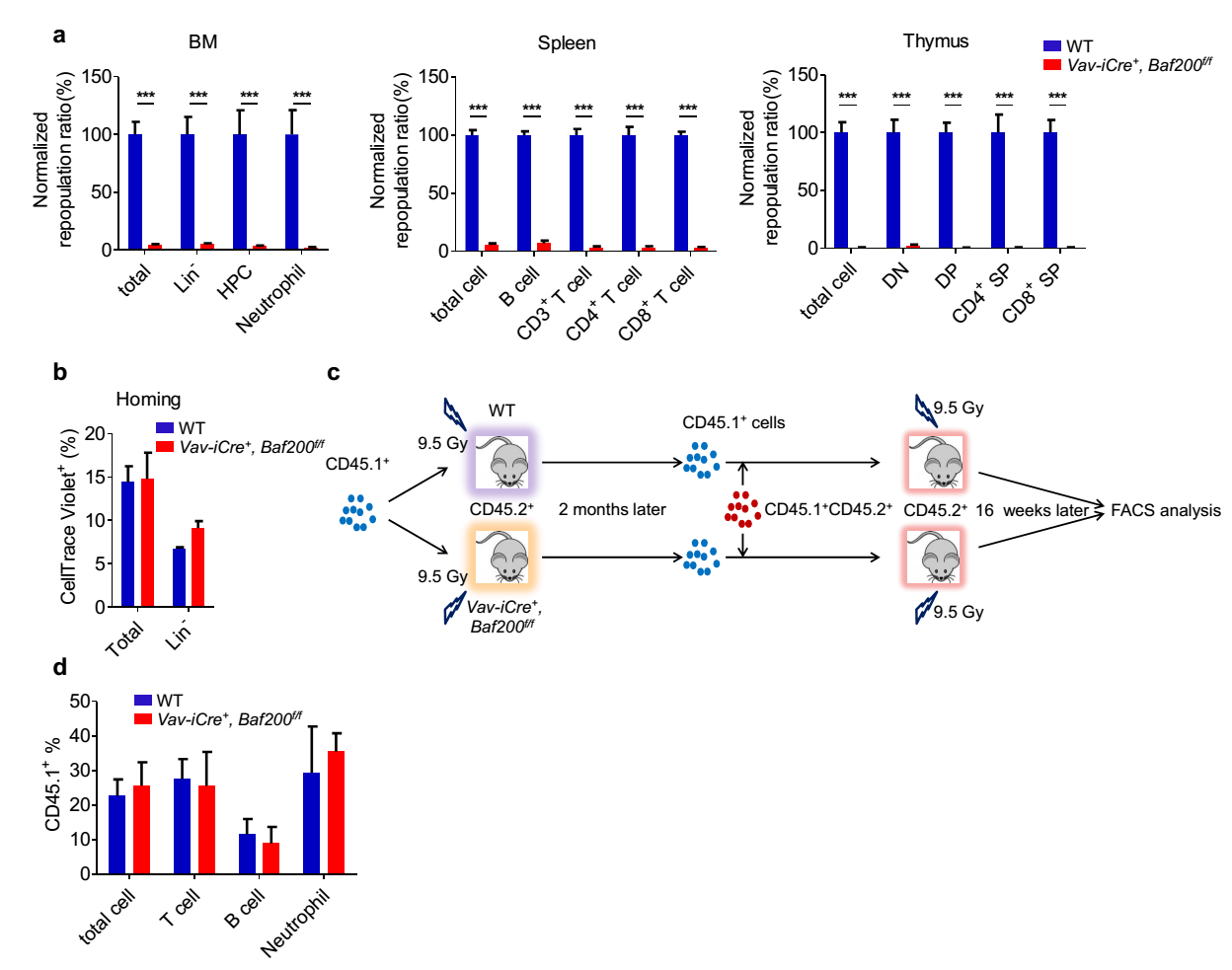


**Figure S9.** Cell-intrinsic role of Baf200 in HSCs function, related to Figure 6. **(a)** Graph showing the relative ratios of CD45.2 versus CD45.1 of the indicated cell types in recipient mice 16 weeks after transplantation, related to Figure 6a (n=6 recipient mice per donor genotype). **(b)** Graph showing the percentage of donor cells in homing assay (n=3 recipient mice per donor genotype). **(c)** Scheme to investigate the impact of microenvironment in *Vav-iCre^+^, Baf200^f/f^* mice. **(d)** Graph showing the percentage of CD45.1^+^ cells in the indicated subsets from recipient mice 16 weeks post-transplantation. There is no difference in engraftment between cells that were exposed to WT or *Vav-iCre^+^, Baf200^f/f^* microenvironment (n=6 recipient mice per donor genotype). Data are shown as means±SEM. ***P<0.001.


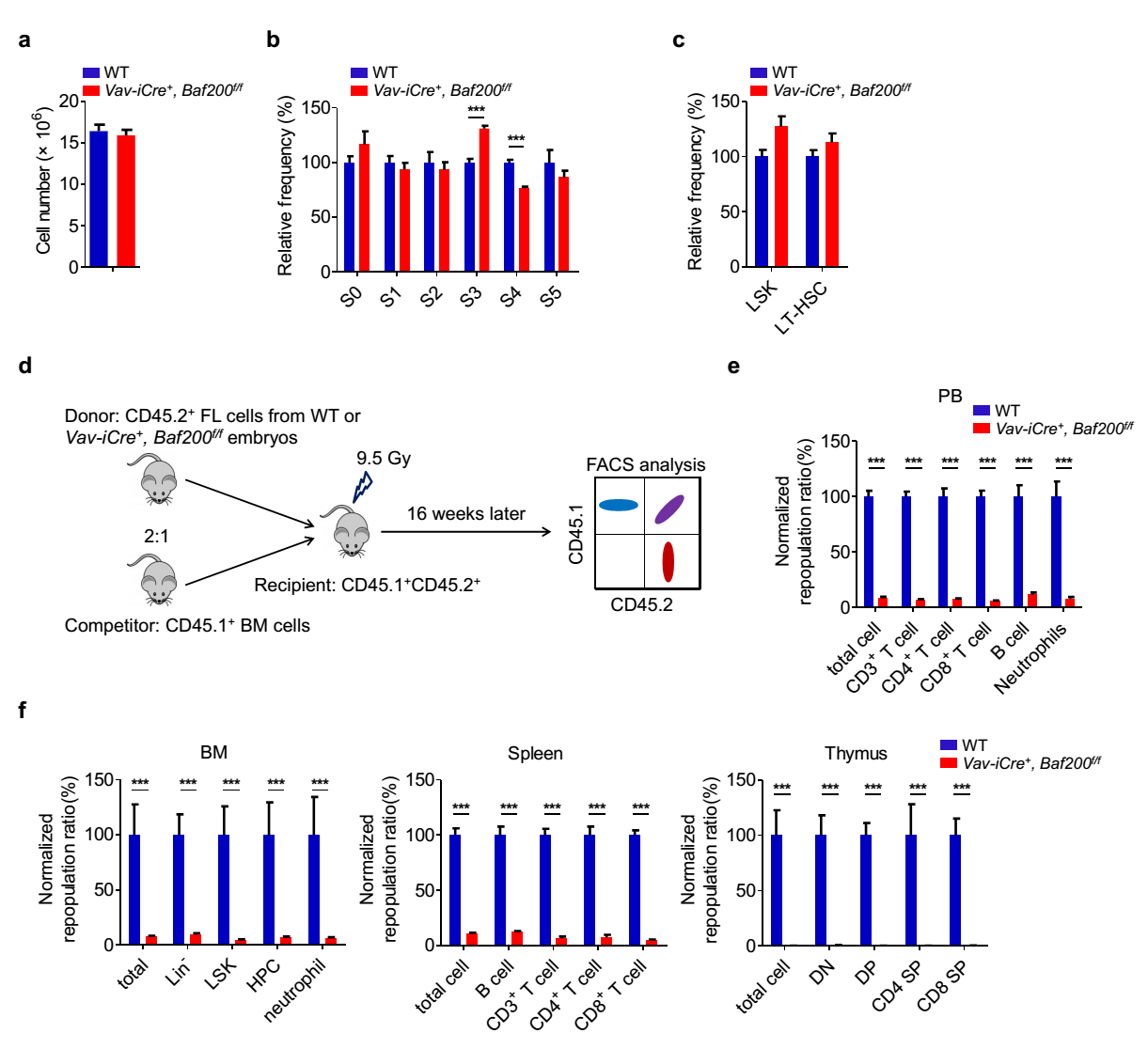


**Figure S10.** FL HSCs from *Vav-iCre^+^, Baf200^f/f^* mice show impaired long-term reconstitution potential. **(a)** Absolute cell number of FLs from E14.5 WT and *Vav-iCre^+^, Baf200^f/f^* embryos (n=10 per genotype). **(b)** Relative frequencies of CD71/Ter119 erythroid subsets from E14.5 WT and *Vav-iCre^+^, Baf200^f/f^* embryos (n=6 per genotype for each subset). **(c)** Relative frequencies of LSK cells and LT-HSCs in the FLs from E14.5 WT and *Vav-iCre^+^, Baf200^f/f^* embryos (n=7-8 per genotype for each subset). **(d)** Scheme of competitive FL transplantation assay. **(e-f)** The relative ratios of CD45.2 versus CD45.1 of the indicated cell types in recipient mice 16 weeks after transplantation (n=4-5 recipient mice per donor genotype). Data are shown as means±SEM. ***P<0.001.
